# Genome Evolution of Bartonellaceae Symbionts of Ants at the Opposite Ends of the Trophic Scale

**DOI:** 10.1093/gbe/evy126

**Published:** 2018-07-02

**Authors:** Gaelle Bisch, Minna-Maria Neuvonen, Naomi E Pierce, Jacob A Russell, Ryuichi Koga, Jon G Sanders, Piotr Łukasik, Siv G E Andersson

**Affiliations:** 1 Cell and Molecular Biology, Science for Life Laboratory, Department of Molecular Evolution, Uppsala University, Sweden; 2 Department of Organismic and Evolutionary Biology, Harvard University; 3 Department of Biology, Drexel University; 4 Bioproduction Research Institute, National Institute of Advanced Industrial Science and Technology, Tsukuba, Japan; 5 Department of Pediatrics, University of California San Diego, La Jolla; 6 Division of Biological Sciences, University of Montana

**Keywords:** ants, bacteria, genomics, molecular evolution, nutritional symbionts

## Abstract

Many insects rely on bacterial symbionts to supply essential amino acids and vitamins that are deficient in their diets, but metabolic comparisons of closely related gut bacteria in insects with different dietary preferences have not been performed. Here, we demonstrate that herbivorous ants of the genus *Dolichoderus* from the Peruvian Amazon host bacteria of the family Bartonellaceae, known for establishing chronic or pathogenic infections in mammals. We detected these bacteria in all studied *Dolichoderus* species, and found that they reside in the midgut wall, that is, the same location as many previously described nutritional endosymbionts of insects. The genomic analysis of four divergent strains infecting different *Dolichoderus* species revealed genes encoding pathways for nitrogen recycling and biosynthesis of several vitamins and all essential amino acids. In contrast, several biosynthetic pathways have been lost, whereas genes for the import and conversion of histidine and arginine to glutamine have been retained in the genome of a closely related gut bacterium of the carnivorous ant *Harpegnathos saltator*. The broad biosynthetic repertoire in Bartonellaceae of herbivorous ants resembled that of gut bacteria of honeybees that likewise feed on carbohydrate-rich diets. Taken together, the broad distribution of Bartonellaceae across *Dolichoderus* ants, their small genome sizes, the specific location within hosts, and the broad biosynthetic capability suggest that these bacteria are nutritional symbionts in herbivorous ants. The results highlight the important role of the host nutritional biology for the genomic evolution of the gut microbiota—and conversely, the importance of the microbiota for the nutrition of hosts.

## Introduction

Bacterial symbionts are common in animals and often play fundamental roles in host biology. Bacteria that associate with hosts can provide nutrition, manipulate reproduction, confer resistance against natural enemies and abiotic stressors such as temperature changes, and influence many other life history traits of their hosts (Moran et al. 2008; Feldhaar 2011). Through these and other effects, symbionts can not only affect the competitive abilities of the hosts in an already established population (Jaenike et al. 2010; Himler et al. 2011), but also provide opportunities for expansions or shifts of the ecological niche. Among the best-known examples of such shifts are found in insects of the order Hemiptera: It is thought that the symbiosis with a bacterium that provided essential amino acids and vitamins deficient in plant sap enabled adaptation to, and subsequently specialization on, this imbalanced diet (Bennett and Moran 2013, 2015). Similarly, a symbiosis between the ancestor of carpenter ants (tribe Camponotini) and a gammaproteobacterium now known as *Blochmannia* has provided these ants with a reliable source of essential nutrients, supplementing their diet that frequently consists of substantial amounts of nitrogen-poor honeydew or extrafloral nectar (Davidson et al. 2003, 2004; Cook and Davidson 2006; Feldhaar et al. 2007). Subsequently, carpenter ants have undergone radiation and are currently one of the most species-rich and most widely distributed groups of ants. 

At the same time, close associations with hosts have dramatically influenced the evolution of the bacterial genome. Most free-living bacteria have relatively large genomes that allow them to respond to a variety of environmental challenges, but as they become associated with hosts, many bacterial genes are lost that are no longer needed in the relatively stable host environment (Moran et al. 2008; Toft and Andersson 2010; McCutcheon and Moran 2012). Extreme cases of genome reduction are observed for bacterial strains that have evolved into obligate mutualists (McCutcheon and Moran 2012; Moran and Bennett 2014). These bacteria often colonize specialized host organs such as bacteriomes or crypts within the gut and codiverge as a result of maternal transmission from one host generation to the next (Dale and Moran 2006; Engel and Moran 2013). Genomes of these bacteria shrink over time, and in some of the oldest associations can be as small as 100–200 kb. Ultimately, the genomes may reach a stage where further gene loss is deleterious to the host, meaning that deteriorating genes need to be replaced by functions encoded by the host or by novel, fresh endosymbionts (Moran et al. 2008; Koga et al. 2013). 

Broad 16S rRNA surveys have revealed that diverse hosts and habitats are colonized by various groups of bacteria that may play significant roles in the host biology. Describing the evolutionary histories and biological characteristics of these bacteria is the key to understanding how many of the diverse and ecologically important groups of eukaryotes have emerged. Here, we introduce a nutritional symbiosis that has not been known to the broader microbiome research community: between ants from the genus *Dolichoderus* and bacteria from the family Bartonellaceae of the Alphaproteobacteria.

*Dolichoderus* (Hymenoptera: Formicidae: Dolichoderinae) is a diverse and widely distributed genus of medium-sized to large ants. They feed primarily at low trophic levels, on plant-based diets known for their nutritional imbalance and incompleteness (Blüthgen et al. 2003; Davidson et al. 2003; Cook and Davidson 2006). Because of this, it was suggested that bacterial nutritional symbionts were likely to play a significant role in their biology (Cook and Davidson 2006; Russell et al. 2009). Microscopy-based studies have identified bacterial masses in mid- and hindgut of some *Dolichoderus* species (Bution et al. 2006), and whereas some sequencing-based studies have reported the presence of potential symbionts (Stoll et al*.* 2007), there has been little data on the identity or functions of these microbes (Russell et al. 2009; Sanders et al. 2017).

Bartonellaceae (Alphaproteobacteria: Rhizobiales) have been primarily known as mammalian pathogens vectored by blood-feeding insects (Dehio 2004; Chomel et al. 2009). The first described species of the genus, *Bartonella bacilliformis*, is a lethal human pathogen and the causative agent of Oroya fever. Other species are adapted to wild and domestic animals where they cause asymptomatic chronic infections. Like other intracellular bacteria, *Bartonella* have reduced genomes compared with their free-living relatives in the Rhizobiales (Alsmark et al. 2004; Boussau et al. 2004; Berglund et al. 2009; Guy et al. 2013). Notably, these genomes encode a diverse set of genes for type IV and V secretion systems that are needed for the infection of endothelial cells and erythrocytes in the mammalian host (Dehio 2008; Vayssier-Taussat et al. 2010). These are thought to have facilitated an explosive radiation and invasion into a broad range of mammalian host species (Berglund et al. 2009; Guy et al. 2013). The insect hosts have so far mostly been viewed as vehicles that transport bacteria between the mammalian hosts.

Recently, divergent species in the Bartonellaceae were identified in honeybees (Kešnerová et al. 2015; Segers et al. 2017) and ants (Russell et al. 2009), thus filling in a phylogenetic gap between the mammalian-infecting *Bartonella* clade and genera such as *Brucella* and *Ochrobactrum* that also infect mammals but are not vector-borne. The Bartonellaceae have been identified by 16S rRNA surveys in a variety of ant taxa feeding at lower trophic levels, and were suggested to provide nutritional benefits, particularly related to nitrogen metabolism (Russell et al. 2009; Anderson et al. 2012; Sanders et al. 2017; Hu et al. 2018). However, Bartonellaceae also infect carnivorous ants, including army ants and certain ponerines (Larson et al. 2014; Neuvonen et al. 2016; Łukasik 2017). The first completely characterized genome of a Bartonellaceae gut symbiont, *Candidatus* Tokpelaia hoelldoblerii, was obtained from a predatory Ponerinae ant *Harpegnathos saltator* (Neuvonen et al. 2016).

Here, we use microscopy, molecular surveys, and comparative genomics to characterize Bartonellaceae infecting herbivorous *Dolichoderus* ants. We show that these bacteria have a metabolic repertoire that differs strikingly from that of *Candidatus* Tokpelaia hoelldobblerii, including a broad spectrum of biosynthetic pathways for essential amino acids that are likely to be deficient in the plant-based diets of the *Dolichoderus* ants (Blüthgen et al. 2003; Davidson et al. 2003; Cook and Davidson 2006). We discuss the implications of these results regarding the roles that these bacteria may play for the ants.

## Materials and Methods

### Sampling and Microscopy

Ant specimens were collected in two locations in Peru: The Centro de Investigaciones y Capacitaciones Rio los Amigos biological research station near Puerto Maldonado (Madre de Dios province, −12.5, −70.1); and the Villa Carmen biological research station near Pillcopata (Cuzco province, −12.89, −71.41—colony JGS2372). Replicate specimens from a single colony were partially dissected and their digestive tracts individually preserved in ethanol or RNAlater (Invitrogen/ThermoFisher Scientific, Waltham, MA), or alternatively fixed for 2 h at room temperature in 4% formaldehyde solution in PBS, then washed with PBS, dehydrated in an ethanol gradient and preserved in 95% ethanol. Based on morphological characteristics, the studied colonies were confidently placed in the genus *Dolichoderus*, but we could not identify them to species level. To survey the microbial communities in the studied colonies, we extracted DNA from gasters of ants surface-sterilized with bleach. Gasters were ground using sterile pestles after flash freezing in liquid nitrogen. For ten colonies from Puerto Maldonado, we dissected midgut walls from two different specimens, individually preserved in RNAlater. Care was taken to remove gut contents and fragments of other tissues. DNA was extracted using the DNeasy Blood and Tissue kit (Qiagen, Hilden, Germany), following a protocol for Gram-positive bacteria.

The dissected and ruptured gut of one specimen from each colony from Puerto Maldonado was imaged in the field following SYBR Green staining as described previously (Sanders et al. 2017). The fixed guts of specimens from colony JGS2372 were rehydrated, carefully dissected, and embedded in resin (Koga et al. 2009; Sanders et al. 2017). Two micrometers of sections were used for hybridization with a combination of fluorescently labeled eubacterial probes EUB338 and EUB897, as well as a probe specific to a subset of Rhizobiales (supplementary table S1, Supplementary Material online), which perfectly matched the sequence of the Bartonellaceae symbiont of that specimen (modified after Thayanukul et al. 2010).

The DNA extracted from dissected midgut walls was used for PCR with universal eubacterial primers 9Fa and 1513R, and the products were sequenced with universal eubacterial primers 789F and 907R (Baker et al. 2003). For 18 of these specimens, we obtained high quality, unambiguous sequences matching Bartonellaceae. For these specimens, we amplified two protein-coding regions by PCR using newly designed primers targeting the *pyrG* and *rpoB* genes (supplementary table S1, Supplementary Material online), based on alignments of these genes for several Bartonellaceae species. Amplicon sequencing libraries were prepared using DNA extracted from ant gasters at Argonne National Laboratory, following Earth Microbiome Project protocols. Data were analyzed using mothur v. 1.37.4 (Schloss et al. 2009) following the protocol described in detail by Łukasik (2017). This included a custom decontamination step, where libraries prepared for “blank” negative samples were used as references for identification of bacterial genotypes derived from reagents and the laboratory environment.

### Genome Sequencing, Assembly, and Annotation

DNA from the *Dolichoderus* spp. symbionts was obtained from dissected midguts of *Dolichoderus* spp. hosts, as described above. Libraries were constructed using the KAPA low-throughput Illumina library preparation kit from extracted DNA sheared on a Covaris S220 sonicator. The libraries were amplified with the KAPA HiFi amplification kit, quality assessed on an Agilent Bioanalyzer, pooled, and sequenced on an Illumina HiSeq2500 instrument at the Harvard Bauer Core Facility.

Reads were assembled using SPAdes 3.1.0 (Bankevich et al. 2012) and CAP3 (Huang and Madan 1999). For each assembly, TBlastX was used to search all contigs against a database consisting of multiple genomes of Bartonellaceae, *Wolbachia*, other bacteria, a fungus, and a Dolichodrinae ant *Linepithema humile* (*E* < e−50). The contigs with top hits to any of these taxa as well as the 10-kb longest contigs with no match below the significant threshold were extracted for analyses of sequence read coverage and GC-content. Contigs within the expected range of read coverage and GC content values, most of which showed significant sequence similarity to Bartonellaceae, were retained in each assembly (supplementary table S2, Supplementary Material online).

Completeness was estimated using the method of Land et al. (2014). Briefly, the completeness score is the average of four different scores: A sequence quality score, which takes into account the number of contigs and gaps in each genome, an rRNA score that assesses the presence of complete ribosomal RNA operons, a tRNA score that assesses the presence of at least one tRNA for each amino acid, and an essential genes score based on the presence of 102 universally conserved genes. Whole genomes of *Ca.* T. hoelldobblerii and of the *Dolichoderus* spp. symbionts were aligned one against each other using MUMmer 3 with the Promer algorithm (Kurtz et al. 2004), using the “maxmatch” option for the alignment and the “layout” and “filter” options while drawing the plots.

Protein-coding genes were predicted using the Prodigal server (Hyatt et al. 2010), rRNA with the RNAmmer server (Lagesen et al. 2007) and tRNA-encoding genes using the tRNA scan server (Lowe and Eddy 1997; Lowe and Chan 2016), all with the standard values. Gene annotations were transferred from *Ca*. T. hoelldobblerii and completed with COGs predictions. For this, the predicted protein sequences were blasted against the COG database (Tatusov et al. 2000, downloaded on March 19, 2015, *e*-value 0.01). CAI, GC%, and GC3% were predicted for each coding sequence using CodonW (http://codonw.sourceforge.net/, last accessed August 1, 2017) with standard parameters, averaged for every contig (supplementary table S2, Supplementary Material online). Intergenic sequences were extracted using Artemis (Rutherford et al. 2000) and blasted against the NCBI nr database (BlastX, *E* < *e*−10). The GC content of intergenic sequences was predicted using a custom perl script. Supplementary figs. S1, S5–S7, Supplementary Material online were made using R (R Core Team 2013). The metabolic pathways of the JSC stains and *Ca*. T. hoelldobblerii were predicted using a Blast Koala search (Kanehisa et al. 2015) against the KEGG species_prokaryotes database (last accessed on March 30, 2017). Autotransporter domains were predicted by blasting the JSC strains proteins against the CDD database (*E* < 0.01) (Marchler-Bauer et al. 2015).

The 16S rRNA identities were calculated from Clustal W2 alignments (Larkin et al. 2007). AAI was chosen instead of average nucleotide identity (ANI) because the latter was too close of the 75% limit of detection. Prodigal-predicted proteomes were compared using the two-way AAI calculator (http://enve-omics.ce.gatech.edu/ani/, last accessed on January 12, 2016) with the standard options: No minimal length, a minimal protein identity of 20%, no minimal score, and a minimal number of alignments of 50.

### Phylogeny and Gene Flux Analyses

The 16S rRNA, *rpoB*, and *pyrG* gene sequences of *Dolichoderus* spp. symbionts and a sample of other ants were sequenced as a part of this study (see supplementary table S1, Supplementary Material online for primers). The 16S rRNA, *rpoB*, and *pyrG* nucleotide sequences of 13 *Bartonella* and 6 outgroup species were retrieved from public databases (supplementary table S3, Supplementary Material online). The sequences were aligned with Mafft (L-INS-I algorithm) (Katoh et al. 2002), and the columns that had more than 50% gaps were trimmed with trimAl (Capella-Gutiérrez et al. 2009). A phylogeny was inferred on the concatenated sequences using RAxML (Stamatakis 2014) with the GTRGAMMAI model and 100 bootstraps.

All-against-all BlastP (Altschul et al. 1990; Camacho et al. 2009) searches were performed on the Prodigal-predicted proteins from the four *Dolichoderus* spp. symbionts, the *Ca*. Tokpelaia hoelldobblerii, 13 *Bartonella* genomes and 6 outgroup Rhizobiales species (supplementary table S3, Supplementary Material online), with an *E*-value cutoff of 10^−3^. The proteins were clustered into families using OrthoMCL (Enright et al. 2002; Li et al. 2003), resulting in a data set of 293 single-copy orthologs. Proteins from each family were aligned using Mafft L-INS-I (Katoh et al. 2002) and alignments were trimmed for all sites with over 50% gaps with trimAl (Capella-Gutiérrez et al. 2009). A phylogeny was then reconstructed from the concatenated protein sequences, using the PROTCATLG model in RAxML (Stamatakis 2014) with 100 bootstap replicates.

A subset of the species used for the OrthoMCL clustering (*B. henselae*, *B. australis*, *B. tamiae* Th309, *Ca*. T. hoelldobblerii, the four *Dolichoderus* spp. symbionts, *O. anthropi* and *B. melitensis*) was selected to represent the lineages in the concatenated protein tree. For each OrthoMCL SCO family, the sequences were aligned using Mafft-L-INS-I (Katoh et al. 2002) and positions with more than 50% gaps were removed with trimAl (Capella-Gutiérrez et al. 2009). Single protein trees were built using RaxML (PROTGAMMAIWAG) (Stamatakis 2014) with 100 bootstrap replicates. Using Newick Utilities (Junier and Zdobnov 2010), all trees were rerooted using *O. anthropi* and *B. melitensis* as outgroups. Only the trees with a bootstrap score ≥70 for all branches and supporting the original phylogeny were selected for further analysis. Using Newick utilities, branch lengths were calculated for the *Dolichoderus* spp. symbionts clade. The relative branch length of the strain JSC161 was calculated by dividing the length of the JSC161 branches with the average length of the other JSC branches.

Trees for UreC, GlnA, HisD, HutU, PdxK, and NadABC were inferred based on alignments of protein sequences retrieved by BlastP searches (Camacho et al. 2009) against NCBI's nr database (*E* < 0.001). A subset of 66 sequences of the *NadA* genes was selected using CD-Hit (Li and Godzik 2006, *c* = 0.8) and the corresponding *NadB* and *NadC* sequences were manually selected. Sequences were aligned with Mafft L-INS-I (Katoh et al. 2002), trimmed for all sites with over 50% gaps with trimAl (Capella-Gutiérrez et al. 2009). The NadABC alignments were concatenated using SeaView (Gouy et al. 2010). Phylogenetic trees were constructed using the PROTGAMMALG model in RAxML (Stamatakis 2014) with 100 bootstap replicates.

The OrthoMCL clustering was used to reconstruct gene gain and losses along the phylogenetic tree. Because genes could be missing from the *Dolichoderus* spp. symbionts’ genomes due to their incomplete status, we wanted to avoid predicting false gene losses. For this, we considered the pan-genome of the four *Dolichoderus* spp. symbionts as a single strain, and kept only one representative gene in each OrthoMCL family. Those clusters were then used to infer gene family losses and gains. Generalized parsimony with ACCTRAN in PAUP* 4.0b10 (Swofford 2002) was used with the following penalties: Gene genesis = 10, gene loss = 5, gene duplication = 2 and all other copy number variation = 0.2 per copy. The changes in the orthologous groups were finally mapped onto the concatenated single-copy orthologs tree.

## Results

### Bartonellaceae Colonize the Midgut Epithelium of *Dolichoderus* spp. Ants

We characterized gut symbioses in eleven colonies of ants from two sites in the Peruvian Amazon. Based on morphological characteristics, the ants from each of these colonies were classified to the genus *Dolichoderus*, but we were not able to identify them to the species level. Our repeated attempts to barcode the specimens yielded clean COI sequences in half of the cases. Voucher specimens have been deposited in the Museum of Comparative Zoology at Harvard pending further characterization. Several different approaches were used for microbial community characterization.

**Fig. 1.**
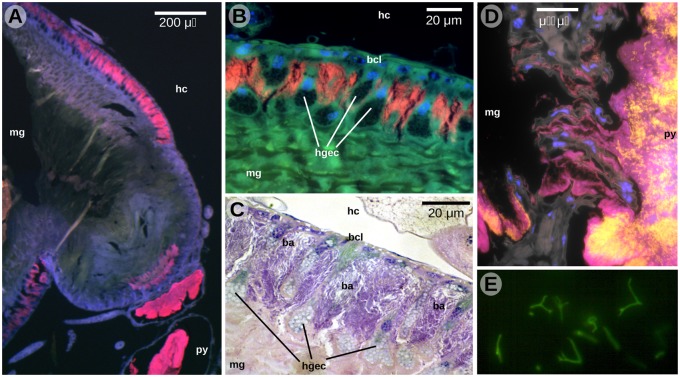
—Localization of bacteria within the digestive tract of *Dolichoderus* sp. JSC2372. (*A*) Cross-section of lower midgut and pylorus. (*B*) Close-up of the midgut wall. (*C*) Close-up of the midgut wall—hematoxylin and eosin staining. (*D*) Transition between midgut and pylorus. (*E*) Isolated cells of a midgut wall colonizer of JSC188, stained with SYBR Green. The field microscope was not calibrated, but the approximate bacterial cell length was 30 µm. In panels a, b, d (resin section FISH), red indicates the signal of eubacterial probes, yellow, Rhizobiales-specific probe; blue, DAPI; green, tissue autofluorescence. hc, hemocoel; mg, midgut; py, pylorus; bcl, basal cell layer; hgec, hypertrophied gut epithelial cells; ba, bacterial cells.

First, we used microscopy to visualize bacteria in the digestive tracts of freshly captured workers from ten colonies from Puerto Maldonado, Madre de Dios Region. In all cases, we observed large numbers of bacterial cells in the ant midgut walls as well as in hindgut lumen (Sanders et al. 2017). The eleventh colony, JSC2372, collected at a later time in Pillcopata, Cusco Region, was characterized more thoroughly. As in the previously studied colonies, fluorescence in situ hybridization (FISH) using universal eubacterial probes (Łukasik 2017) revealed abundant bacteria in the midgut wall and hindgut lumen (fig. 1*A*). Imaging at higher magnifications showed that rod-shaped bacteria colonized the cavities between the hypertrophied midgut epithelial cells (figs. 1*B* and 1*C*). In this tissue, the signal of the eubacterial probes overlapped perfectly with the signal of a probe specific to bacterial order Rhizobiales, suggesting that most if not all bacteria in the midgut wall are members of that order. Bacteria were also observed in the lumen of the pylorus and hindgut, and approximately half of them stained with the Rhizobiales-specific probe (fig. 1*D*). Interestingly, our early observations of SYBR Green-stained bacterial cells from the midgut wall revealed their unusual, branched structures (fig. 1*E*).

Second, we estimated the bacterial abundance in the JSC085, JSC188, and JSC189 colonies using previously published data on gut bacterial densities in rainforests ants obtained via qPCR targeting the 16S rRNA genes (Sanders et al. 2017). After normalizing for the DNA concentration as a proxy for total biomass, we estimated the gut bacterial abundance to range from 1,404 copies/pg in JSC085 to 4,376 copies/pg in JSC188 (supplementary fig. S1, Supplementary Material online). These estimates are comparable to the median value of 2,257 pg/DNA for a larger set of arboreal Peruvian *Dolichoderus* genera, and at the upper range of bacterial abundances across a large collection of ants (Sanders et al*.* 2017). 

Third, we performed amplicon sequencing of the 16S rRNA gene using DNA extracted from the surface-sterilized gaster (the posterior, bulbous part of the ant body, containing most of the digestive tract) of a single worker from each of the eleven colonies (supplementary fig. S2, Supplementary Material online). The *Dolichoderus* gaster amplicon libraries were dominated by a relatively small number of bacterial taxa that showed sequence similarity to previously characterized species of Bartonellaceae, Entomoplasmatales, *Wolbachia*, Burkholderiales, Xanthomonadales, Pseudomonadales, and Sphingobacteriales species (supplementary fig. S2*A*, Supplementary Material online). Of these, sequences showing similarity to Bartonellaceae were by far the most abundant in the data set, although the communities of two of the ants were dominated by an Entomoplasmatales bacterium (supplementary fig. S2*A*, Supplementary Material online). The bacteria of the Bartonellaceae in the *Dolichoderus* ants were represented by four distinct 97% OTUs; the median relative abundance for the four OTUs combined was 87.8% (range 0.4–100%). Most specimens hosted several distinct genotypes of Bartonellaceae, often from more than one OTU (supplementary fig. S2*B*, Supplementary Material online).

Fourth, we carefully dissected midgut walls of two workers from each of the ten colonies from Puerto Maldonado, which had been preserved in RNAlater in the field and kept frozen until dissection. Extracted DNA was used as a template for amplification and Sanger sequencing of the nearly full-length 16S rRNA gene using universal eubacterial primers. In 18 out of 20 cases, we obtained high-quality sequences matching Bartonellaceae. In all cases, these Sanger sequences were identical in the two specimens from the same colony, and perfectly matched the most abundant genotype in our 16S rRNA amplicon data set, obtained from the third specimen from the same colony (supplementary fig. S2*B*, Supplementary Material online). Together, these data reveal that Bartonellaceae from a larger ant-specific lineage (Russell et al. 2009) are the dominant gut community members of Peruvian *Dolichoderus* ants, and that they primarily inhabit the ant midgut epithelium.

### Genome Characteristics of the *Dolichoderus* spp. Symbionts

We used DNA extracted from the dissected midgut wall of a single worker from each of colonies JSC085, JSC161, JSC188, and JSC189 for metagenomic library preparation and sequencing. In all cases, the metagenome assemblies contained a set of high-coverage contigs that showed significant sequence similarity (*E* < *e*−50) to the genomes of the recently characterized gut symbiont of a predatory ant *H.saltator* named *Ca.* T. hoelldobblerii (Bartonellaceae) (Neuvonen et al. 2016), and to diverse species and strains from the genus *Bartonella* (supplementary fig. S3, Supplementary Material online). Contigs that corresponded to the nuclear and mitochondrial genomes of the host ant as well as contigs with lower read coverage and/or different GC content values were not included in the final symbiont genome assemblies.

We obtained from 3 to 39 assembled contigs for each strain (supplementary table S2, Supplementary Material online) that summed up to genomes of 0.96 Mb (JSC161) to 1.53 Mb (JSC189), putatively encoding 900–1,300 proteins, respectively (supplementary table S4, Supplementary Material online). The genomic GC content values ranged from 38.5% to 43%, with the smallest genome of JSC161 being the most AT-rich (supplementary table S4). Gene order was not conserved among the four Bartonellaceae genomes, not even in comparison with the two largest contigs of >700 kb in the JSC085 and JSC161 strains, thus indicating extensive genome shuffling (supplementary fig. S4, Supplementary Material online). With genomes of around 1 Mb, the JSC085 and the JSC161 strains represent the smallest genomes identified thus far in the Bartonellaceae.

The genomes were estimated to be 92–98% complete according to the method of Land et al. (2014), with the smallest genome of the JSC161 strain being the most complete, and the largest, and most fragmented, genome of the JSC189 strain the least complete. We considered the possibility that the smallest genome assembly might contain too few contigs. However, given that the 3 contigs in the JSC085 genome have a more than 10-fold higher read coverage than most other contigs and that no contigs of similar GC content and coverage were identified, it is unlikely that the JSC085 genome is larger than the estimated 1.1 Mb. In the case of the JSC161 genome, a midcoverage contig of 14 kb showed best BLAST hits to *Bartonella*, but due to 3- to 10-fold lower coverage this contig was not included in the final assembly.

Vice versa, the largest genome assemblies might contain too many contigs. We confirmed that contigs of >20 kb in the final genome assemblies showed best matches to *Bartonella* or *Ca.* Tokpelaia hoelldobblerii (supplementary table S2, Supplementary Material online). We are thus confident that these contigs belong to the Bartonellaceae genomes. However, some of the smaller contigs <20 kb showed sequence similarity to mobile elements and were associated with mean GC content values at third codon synonymous sites (GC3s) that deviated from the overall mean (supplementary fig. S5 and table S2, Supplementary Material online). For example, the JSC188 assembly (mean GC3s =30%) contained two smaller contigs of 5.7 and 20.5 kb with GC3s values of 34.5% and 37.4%, respectively, of which one showed sequence similarity to a cryptic plasmid in *Bartonella grahamii*. Although most of the genes on these smaller contigs also showed significant BLAST hits to genomes of *Bartonella* spp., *Ca*. Tokpelaia hoelldobblerii or other members of the Rhizobiales, we cannot show conclusively that they belong to the *Dolichoderus* spp. symbionts genomes. However, even with their inclusion, the total size of the JSC189 genome does not exceed 1.5 Mb. The assembly from specimen JSC189 also contained a number of low-coverage contigs showing homology to Bartonellaceae genomes (supplementary fig. S5, Supplementary Material online). These contigs may represent a related strain present at a more than 100-fold lower abundance (supplementary fig. S3, Supplementary Material online) possibly derived from a contaminant in the hindgut (fig. 1). These low-abundance genomes might be contributing to the fragmented nature of the JSC189 assembly.

Interestingly, the small JSC161 genome was also the most gene dense genome, with a coding density of 91% compared with only 75% for the largest genome. A plot of the size distribution of the intergenic regions in contigs of >20 kb confirmed a relatively higher fraction of long spacers in the larger genomes (supplementary fig. S6, Supplementary Material online). In the smallest genome, more than 40% of spacers were <50 bp and <20% were more than 200 bp. In the largest genome, these proportions were reversed such that only 20% were <50 bp, whereas more than 40% of the spacers were longer than 200 bp. The two largest assembled contigs of the JSC188 and JSC189 strains contained as many as 47 and 90 intergenic regions, respectively, with sizes of more than 1 kb (supplementary fig. S7, Supplementary Material online). A BlastX search of these intergenic regions >1 kb against the NCBI nr database yielded several strong hits (*E* < *e*^−10^) (supplementary table S5, Supplementary Material online), revealing gene remnants with frameshift mutations and/or small deletions.

### The Ant-Associated Lineages are Monophyletic

**Fig. 2.**
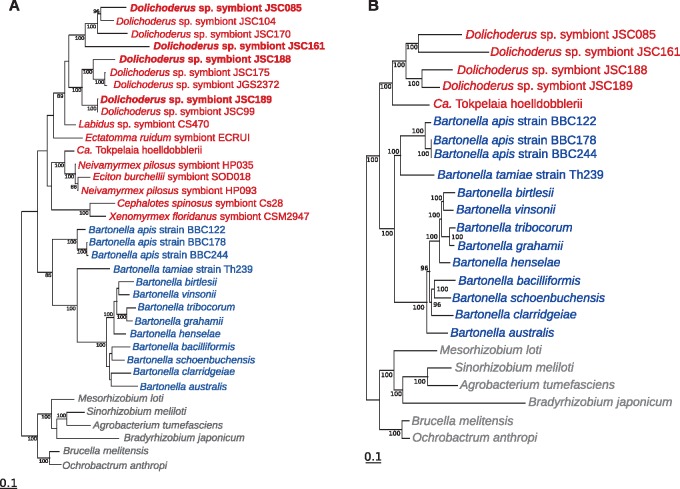
—Relationships among Bartonellaceae from ants and other hosts. Phylogenetic trees were inferred based on (*A*) a concatenated alignment of partial 16S, *rpoB* and *pyrG* nucleotide sequences and (*B*) a data set of 293 concatenated protein sequences. Only bootstrap values >80% are shown. Ant symbionts are shown in red color; *Bartonella* species in blue, and outgroups in gray. *Tokpelaia* strains with sequenced genomes are indicated in bold.

In order to understand the relationships among symbionts of *Dolichoderus* spp., associates of other ants, and other members of the Bartonellaceae, we amplified and compared partial sequences of three genes, *rrs*, *rpoB*, and *pyrG* coding for 16S rRNA, RNA polymerase subunit B and CTP synthase from the total of 17 ant-associated strains. A maximum likelihood phylogeny based on concatenated partial sequences of the three genes (fig. 2*A*) confirmed prior reports (Russell et al. 2009) that the ant-associated strains form a monophyletic clade, which is sister to the genus *Bartonella* comprising insect-vectored mammalian bacteria and gut bacteria of honeybees (Kešnerová et al. 2015; Segers et al. 2017). Furthermore, the phylogeny indicated that symbionts of *Dolichoderus* spp. form two well-supported clades. Strains JSC085 and JSC161 belonged to one of these clades, and strains JSC188 and JSC189 belonged to the other. Unfortunately, because of relatively scarce sampling of the diverse Bartonellaceae strains from other ants (Russell et al. 2009; Sanders et al. 2017), we could not draw conclusions on the monophyly of all four *Dolichoderus* spp. symbionts relative to strains from other ants. Nevertheless, *Ca*. T. hoelldobblerii clearly was not part of either of the two well-supported *Dolichoderus* clades, but seemed closer to strains that infected some specimens in predatory army ants of the genera *Neivamyrmex* and *Eciton* (Łukasik 2017).

The maximum likelihood phylogeny based on a concatenated alignment of 293 proteins (supplementary table S6, Supplementary Material online) encoded by single-copy genes in strains for which genome data are available (fig. 2*B*) recapitulated the relationships inferred based on three genes for a larger set of ant-associated strains. The tree topology confirmed that the ant-associated bacterial clade is a sister group to the *B. apis* of honeybees and the mammalian-associated *Bartonella* species with 100% bootstrap support (Neuvonen et al. 2016; Segers et al. 2017). Importantly, the genome-based tree showed that the four *Dolichoderus* spp. symbionts clustered together with *Ca.* T. hoelldobblerii with 100% bootstrap support. Thus, all the ant-associated bacteria with sequenced genomes are monophyletic.

The JSC161 strain was placed on a long branch in both the nucleotide and the protein tree, suggesting that it has experienced an increase in the rate of sequence evolution. To assess whether the evolutionary rate of the JSC161 lineage was inflated due to a small subset of rapidly evolving or pseudogenized genes, we estimated the relative branch lengths in 73 single protein trees with a topology that matched the concatenated tree topology with >70% support for each node. The branch lengths to the JSC161 lineage from its divergence node showed a normal length distribution (supplementary fig. S8, Supplementary Material online), indicating that the majority of genes in the genome have been equally affected by the increased rate of sequence evolution.

To quantify the relatedness of the *Dolichoderus* spp. symbionts and *Ca.* T. hoelldobblerii, we calculated the 16S rRNA gene identities as well as the average amino acid identities (AAI) (supplementary table S7, Supplementary Material online). The 16S rRNA genes of the *Dolichoderus* symbiont strains shared 95–96% identity with the 16S rRNA gene of *Ca*. T. hoelldobblerii, suggesting that they should be classified into the same genus. Consistently, the AAI values in all pairwise comparisons with *Ca*. T. hoelldobblerii were 55% or higher, also supporting the hypothesis that they belong to the same genus (Rodriguez and Konstantinidis 2014). These levels of divergence could justify classifying *Dolichoderus* spp. symbiont strains as different species of the genus *Tokpelaia*. However, the naming convention in the field of insect endosymbiosis has been to keep the species names constant for bacterial strains that may infect different species of host insects, as long as they result from a single ancestral infection followed by vertical transmission (Husnik and McCutcheon 2016). Thus, until the biology and phylogenetic relationships of the *Dolichoderus* spp. symbionts are further clarified, we conservatively propose to classify them as strains of a single species, *Candidatus* Tokpelaia dolichoderi.

### Inferences of Gene Gains and Losses in the Ant-Associated Bacterial Genomes

**Fig. 3.**
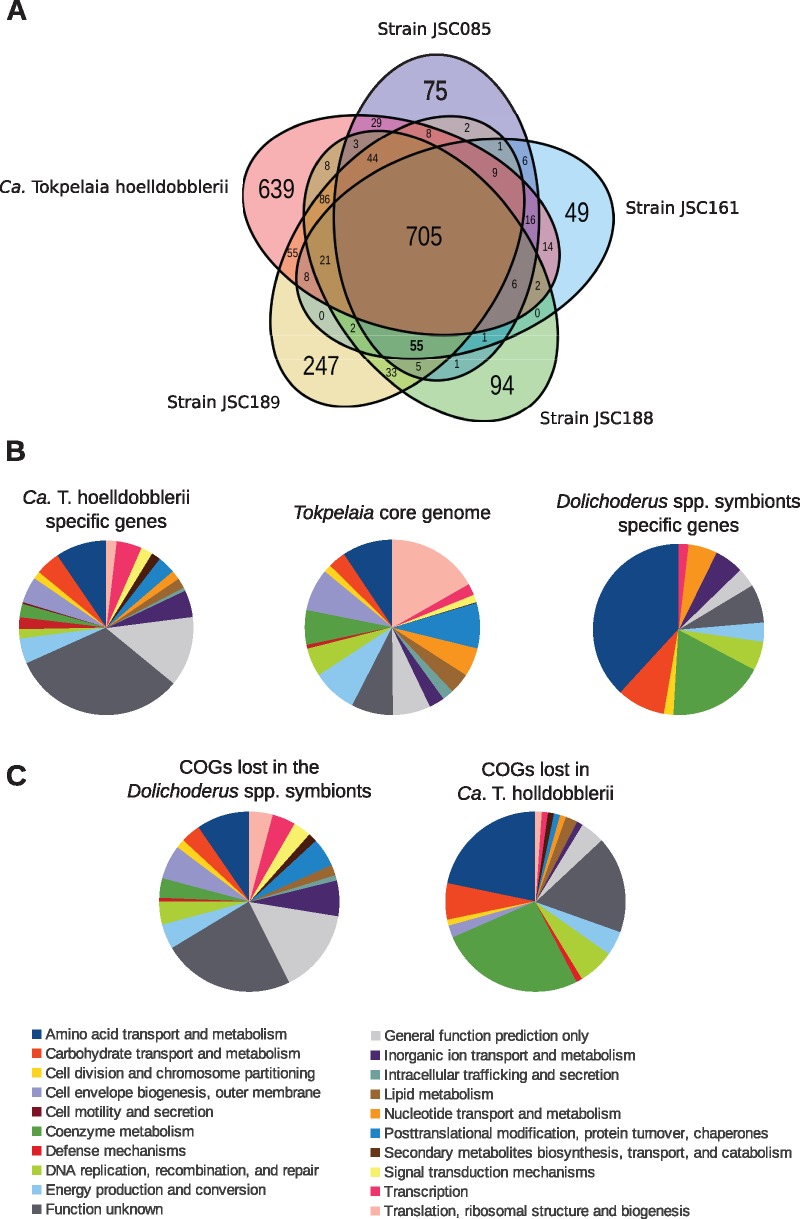
—Comparative genomics of the ant-associated *Tokpelaia* species. (*A*) Diagram showing the number of unique genes in each species as well as the overlaps between the *Dolichoderus* spp. symbionts as well as the overlap with *Ca.* T. hoelldobblerii. The relative fraction of genes sorted into the functional categories is shown for protein families (*B*) that represent the *Tokpelaia* core genome, are unique to *Ca*. T. hoelldobblerri and the *Dolichoderus* spp. symbionts, respectively, and **(*C*)** that have been lost in the *Dolichoderus* spp. symbionts and *Ca*. T. hoelldobblerii, respectively.

To quantify the difference in gene content between the *Dolichoderus* spp. and the *Harpegnathos* symbionts, we clustered all genes into homologous protein families and examined the taxonomic distribution of genes within each family (fig. 3*A*; supplementary table S8, Supplementary Material online). We identified 705 core genes present in both the genome of *Ca*. T. hoelldobblerii and the genomes of the four *Dolichoderus* symbionts. In addition, 639 genes were uniquely present in the genome of *Ca*. T. hoelldobblerii, whereas 55 genes were identified in all the four genomes of the *Dolichoderus* spp. symbionts but not in *Ca*. T. hoelldobblerii. The species-specific genes ranged from 49 in the JSC161 strain, having the smallest genome, to 247 in the JSC189 strain with the largest genome, several of which code for mobile or phage-related elements (supplementary table S8D, Supplementary Material online).

To infer the patterns of gene gains and losses, we performed a gene flux analysis in which we mapped the occurrence patterns of protein families onto the phylogenetic tree using generalized parsimony (supplementary fig. S9, Supplementary Material online). We used two slightly different approaches; in the first analysis each genome represented a distinct taxon (supplementary fig. S9A, Supplementary Material online). However, because the *Dolichoderus* spp. symbionts genomes may not be complete, some genes may incorrectly be inferred as lost in the individual genomes. To address this problem, we combined the four *Dolichoderus* spp. symbionts genomes into a single taxon (supplementary fig. S9B, Supplementary Material online). Because it is unlikely that the same genes got independently lost from each of the four genomes, the latter analysis should provide a reliable estimate of gene loss in the common ancestor of the *Dolichoderus* spp. symbionts, although the number of gained genes is overestimated because they represent the combined sum of the species-specific genes in all four genomes. Based on these results, we estimate that 310 protein families (∼19%) were lost in the common ancestor of the *Dolichoderus* spp. symbionts, whereas 92 protein families (∼5%) were lost in the ancestor of *Ca*. T. hoelldobblerii.

We sorted the protein families uniquely present in the *Dolichoderus* spp. symbionts and *Ca.* T. hoelldobblerii, respectively, into functional categories (supplementary table S8, Supplementary Material online). We examined the functions of the 310 and 92 genes inferred to have been lost in the ancestors of the *Dolichoderus* spp. symbionts and *Ca.* T. hoelldobblerii, respectively (supplementary table S9, Supplementary Material online). A comparison of the results showed that functional categories associated with the metabolism and transport of amino acids and cofactors were highly overrepresented among both the *Dolichoderus* spp. symbionts-specific gene set and the genes lost in *Ca.* T. hoelldobblerii (figs. 3*B* and 3*C*). A closer inspection confirmed that the genes specific to the *Dolichoderus* spp. symbionts were largely lost in *Ca*. T. hoelldobblerii (supplementary tables S8F and S9D, Supplementary Material online). Thus, the genes solely identified in the *Dolichoderus* spp. symbionts appear to be have been present in the ancestor of the Bartonellaceae and lost in the ancestor of *Ca.* T. hoelldobblerii, but conserved in the *Dolichoderus* spp. symbionts.

We also examined the function of the 49 genes that are missing in the JSC161 genome, but present in the genomes of the other three symbionts (supplementary table S9, Supplementary Material online). Of these, 14% are involved in DNA replication, recombination and repair (including *recF*, *recO*, and *recR* in the RecFOR pathway and *recG*), 12% in post-translational modification (including chaperones), 8% in cell envelope biogenesis and 8% in ion metabolism.

### Nitrogen Recycling is a Shared Feature of all Ant Symbionts

It was hypothesized that symbionts of herbivorous ants may facilitate the recycling of nitrogen waste products by degrading urea (Cook and Davidson 2006; Feldhaar et al. 2007). Indeed, in the genomes of all *Dolichoderus* spp. symbionts we identified the *ureABCDEFGJ* operon for the urease subunits and the associated *glnA* gene for glutamine synthetase, consistent with their previous identification in the genome of *Ca*. T. hoelldobblerii (Neuvonen et al. 2016). Phylogenetic analysis based on the UreC protein sequences (supplementary fig. S10*A*, Supplementary Material online) showed that the ant symbionts and *B. apis* are related to most other members of the Rhizobiales. The genes for the glutamine synthetase are also present in the classical *Bartonella* species, and again the phylogeny showed a close relationship with members of the Rhizobiales (supplementary fig. S10*B*, Supplementary Material online), thus confirming that the genes for nitrogen recycling were ancestrally present in the Bartonellaceae, and have been retained in many extant strains.

### The Metabolic Capabilities of the Symbionts Correlate with the Diet of the Ants

To test the hypothesis that the *Dolichoderus* spp. symbionts provide amino acids to the ants, we examined the amino acid biosynthetic repertoires of their genomes. In all *Dolichoderus* spp. symbionts genomes, we identified complete pathways for the biosynthesis of 18 amino acids, including all essential amino acids and all but 2 nonessential amino acids (supplementary table S10, Supplementary Material online). The only genes that we could not identify were the *iscS* gene for the synthesis of Alanine and the *asnB* gene for the synthesis of Asparagine. Thus, the *Dolichoderus* spp. symbionts are able to synthesize all essential amino acids.

**Fig. 4.**
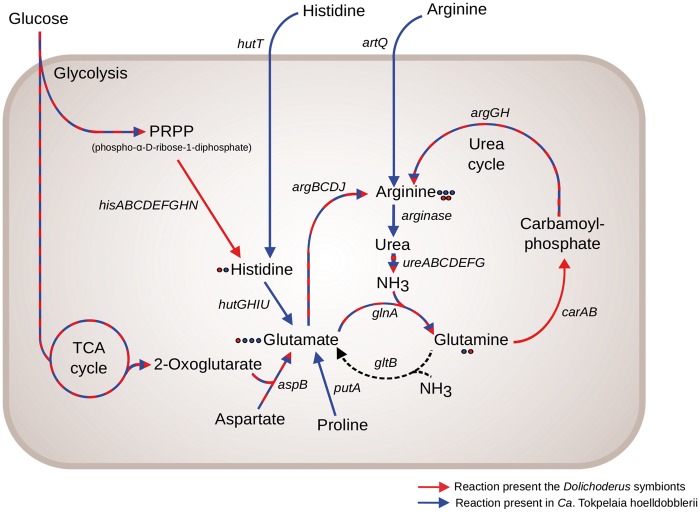
—Biosynthesis and degradation of histidine and arginine in the ant-associated *Tokpelaia* species. Schematic illustration of the transporters and enzymes involved in the import, synthesis and degradation of histidine and arginine. Red arrows indicate presence in the *Dolichoderus* spp. symbionts; blue arrows indicate presence in *Ca*. T. hoelldobblerii. Red and blue dots indicate the number of metabolic pathways that result in a given product for, respectively, the *Dolichoderus* spp. symbionts and *Ca*. T. hoelldobblerii.

**Fig. 5.**
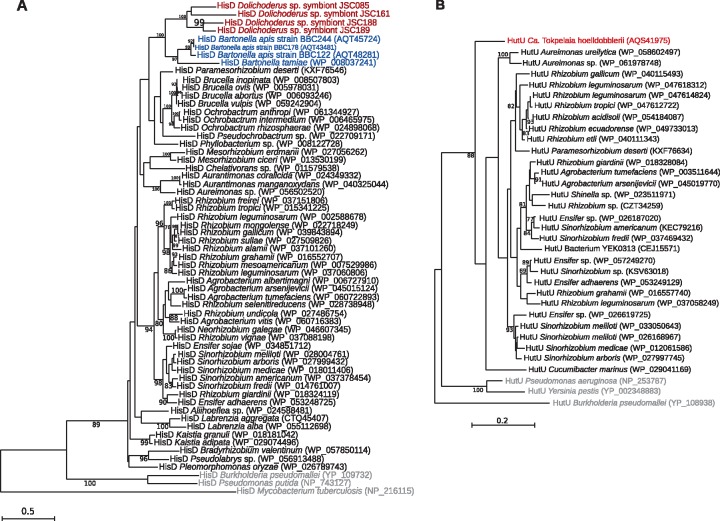
—Phylogeny of proteins involved in the histidine pathways. Phylogenetic trees were inferred based on (*A*) the HisD protein involved in the synthesis of histidine and (*B*) the HutU protein involved in the degradation of histidine. Ant symbionts are shown in red color; *Bartonella* species in blue, and outgroups in gray.

A comparison to *Ca.* T. hoelldobblerii revealed major differences in the pathways related to the biosynthesis of histidine and arginine, which are connected to the urea cycle. The *Dolichoderas* genomes contain the *hisABCDEFGHN* genes for the synthesis of histidine, and the *argBCDJ* and the *carAB* +*argGH* genes for the synthesis of arginine from glutamate and glutamine, respectively (fig. 4). A phylogenetic inference based on the *hisD* gene product, which is the last enzyme in the histidine biosynthetic pathway, showed that the *Dolichoderus* spp. symbionts are related to *Brucella* and *Ochrobactrum*, albeit not with significant bootstrap support (fig. 5*A*). Most species of the Rhizobiales contain a single copy of this gene. The tree topology is thus consistent with the hypothesis that the pathway for histidine biosynthesis was ancestrally present and has been lost independently in *Ca.* T. hoelldobblerii and the classical *Bartonella* species.

Instead, we identified in *Ca*. T. hoelldobblerii the *hutT* gene for an ABC transporter involved in the import of histidine along with the *hutGHIU* genes for enzymes involved the conversion of histidine to glutamate (fig. 4). Additionally, the *Ca.* T. hoelldobblerii genome contained the *artQ* gene for an ABC transporter involved in the import of arginine and a gene for arginase involved in the breakdown of arginine to urea. The *hut* genes could not be identified in any species of the Bartonellaceae other than *Ca.* T. hoelldobblerii, and a phylogeny based on the HutU protein placed *Ca.* T. hoelldobblerii rather distantly from other members of the Rhizobiales (fig. 5*B*). Moreover, only a few species of the Rhizobiales contained the *hut* genes and some of these species contained multiple paralogous genes located on megareplicons, which are prone to gene gains and losses. The multiple gene copies in only a few species indicate that the *hut* genes coding for enzymes involved in the conversion of histidine to glutamine may have evolved through horizontal gene transfers and duplications. Thus, the metabolic profiles suggest that the *Dolichoderus* spp. symbionts are able to synthesize histidine and arginine, essential amino acids deficient in plant-based diets. In contrast, *Ca*. T. hoelldobblerii imports these amino acids from the host and then converts them to glutamine.

The loss of amino acid biosynthetic genes in *Ca.* T. hoelldobblerii also included *thrA* for the biosynthesis of threonine and *aroE*, which code for a key enzyme in the biosynthesis of the aromatic amino acids phenylalanine, tryptophan and tyrosine, suggesting that these amino acids may also be obtained from the host. Indeed, genes for proteases, tripeptidases and dipeptidases as well as deaminases and dehydrogenases were identified in *Ca.* T. hoelldobbleri, but not in the *Dolichoderus* spp. symbionts. Additionally, we identified a gene for the threonine efflux protein RhtB, which represents a conserved transport family for the excretion of homoserine and other amino acids. We hypothesize that the losses of genes for amino acid biosynthetic functions and the retention or acquisition of genes for the degradation of amino acids and peptides is related to the protein-rich diet of *H. saltator*.

**Fig. 6.**
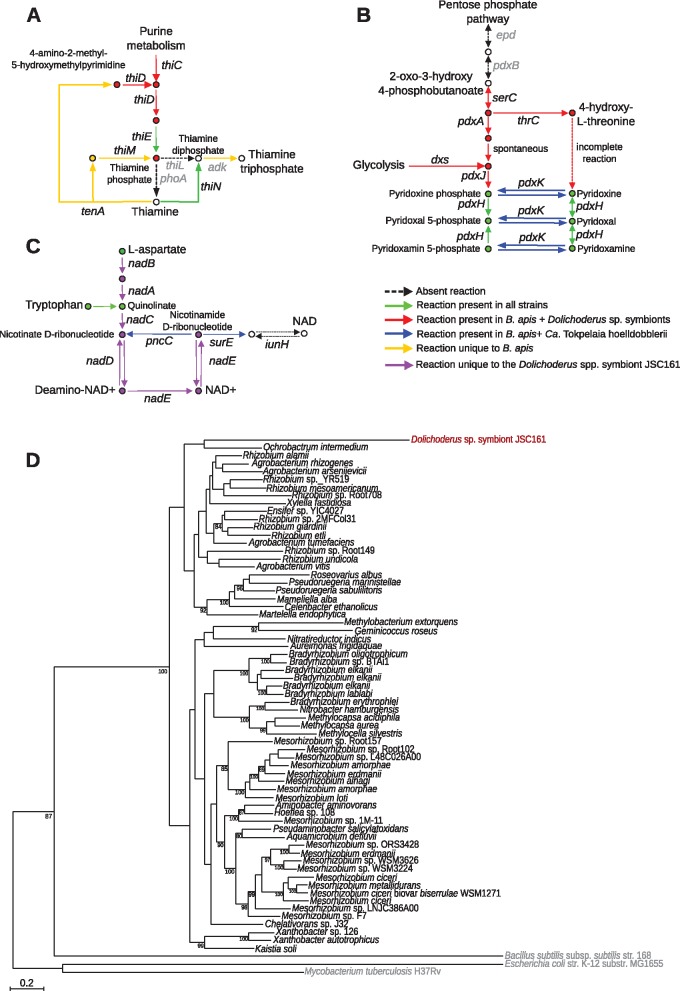
—Vitamin biosynthetic pathways in the ant-associated *Tokpelaia* species. Schematic illustration of genes for the synthesis of vitamins **(***A*) thiamine (B1), (*B*) pyridoxal (B6), and (*C*) nicotinamide (B3). (*D*) Phylogenetic tree inferred from a concatenated alignment of the NadABC genes using the maximum likelihood method. Only bootstrap values above 80% are shown. Red arrows indicate presence in the *Dolichoderus* spp. symbionts; blue arrows indicate presence in *Ca*. T. hoelldobbleri; dashed black arrows and gene names in grey indicate absence. Green dots show the active forms of the vitamins.

The *Dolichoderus* spp. symbionts displayed a broad biosynthetic capability of vitamins, with some strain-specific variability. In all genomes, we identified complete pathways for the biosynthesis of vitamins B2 and B6 (pyridoxine), and partial pathways for the biosynthesis of vitamins B1 (thiamine), B7, and B9 (fig. 6). In the vitamin B1 pathway, the *thiN* gene for the conversion of thiamine into thiamine diphosphate was present in all members of the Bartonellaceae (fig. 6*A*). Additionally, the *Dolichoderus* spp. symbionts contained the *thiCDE* genes for the conversion of purines to thiamine phosphate. A paralog to the *thiE* gene was identified in *Ca.* T. hoelldobblerii as well as in all *Bartonella* genomes. However, several other genes in these pathways were inferred to have been lost from this genome.

In the vitamin B6 pathway for pyridoxine biosynthesis (fig. 6*B*), the *Dolichoderus* spp. symbionts genomes contained the *pdxA* and *pdxJ* genes for the synthesis of pyridoxine phosphate. A phylogeny of the *pdxJ* gene product showed that all *Dolichoderus* spp. symbionts belong to the same clade (with 89% bootstrap support) (supplementary fig. S11*A*, Supplementary Material online). In contrast, a phylogeny of the *pdxA* gene product showed that they segregate into two distinct clades; the JSC085 and JSC161 strains clustered with the classical *Bartonella* species and *B. apis*, whereas the JSC188 and JSC189 strains belonged to a different clade that also contained a paralog in *B. apis* (supplementary fig. S11*B*, Supplementary Material online). We infer that two paralogs of the *pdxA* genes were ancestrally present and differentially lost in the two groups of *Dolichoderus* spp. symbionts. No homolog of these genes could be identified in *Ca.* T. hoelldobblerii, and the sole gene involved in vitamin B6 biosynthesis in this species was *pdxK*, which was related to homologs in *B. apis* and other members of the Rhizobiales (supplementary fig. S11*C*, Supplementary Material online).

Among the species-specific genes, we identified a complete set of genes for the synthesis of vitamin B3 (nicotinamide) in the JSC161 genome (fig. 6*C*), whereas the JSC085 and JSC161 genomes encoded a partial pantothenate pathway for the biosynthesis of vitamin B5, and the JSC188 genome encoded a partial cobalamin pathway for vitamin B12 (supplementary table S10, Supplementary Material online). The presence of a complete pathway for the synthesis of vitamin B3 in the smallest genome, but not in any of the larger *Dolichoderus* spp. symbiont genomes, was surprising. Three of the genes, *nadABC*, were colocated in the genome, whereas the *nadDE* genes were located elsewhere. Homologs of the *nadABC* genes could not be identified in the classical *Bartonella* species, although they were present in the genomes of other members of the Rhizobiales. A phylogeny inferred from a concatenated alignment of the NadABC proteins showed that the JSC161 strain clusters with the other Rhizobiales sequences with 100% bootstrap support, however, this lineage is placed on an exceptionally long branch (fig. 6*D*). Because there are no relatives in any of the *Bartonella* species and no closest relatives within the Rhizobiales species either, we cannot quantify the rate acceleration for these genes, nor can we identify the putative donor species.

Differences between the two groups of ant-associated bacteria were also observed for enzymes involved in the degradation of carbohydrates. Consistent with a sugar-rich diet of the *Dolichoderus* ants, their symbionts have retained both the glycolytic and the Entner–Dudouroff pathway for the production of pyruvate and ATP from glucose and glucose-6-phosphate, respectively. Both pathways were inferred to be ancestrally present, but *Ca*. T. hoelldobblerii has lost the Entner–Dudouroff pathway, whereas the classical *Bartonella* species have lost the glycolytic pathway. The retention of two pathways for the degradation of carbohydrates in the *Dolichoderus* spp. symbionts may be due to the diet of these herbivorous ants, which typically includes large quantities of sugary secretions from hemipterans and plants (Davidson et al. 2004; Cook and Davidson 2006).

### Outer Surface Structures and Secretion Systems

Like extracellular gut bacteria, but unlike many endosymbionts, the Bartonellacee symbionts of ants have not shed their cell wall. The core genome of these bacteria contains a complete set of genes for peptidoglycan and lipopolysaccharide biosynthesis (supplementary table S11, Supplementary Material online). However, we did not identify genes for specific outer surface structures, such as the BadA adhesin or the type IV secretion systems, required for the invasion of endothelial cells and erythrocytes, respectively, in mammal-infecting *Bartonella*. This is consistent with the hypothesis that the ant symbionts do not infect mammalian hosts.

**Fig. 7.**
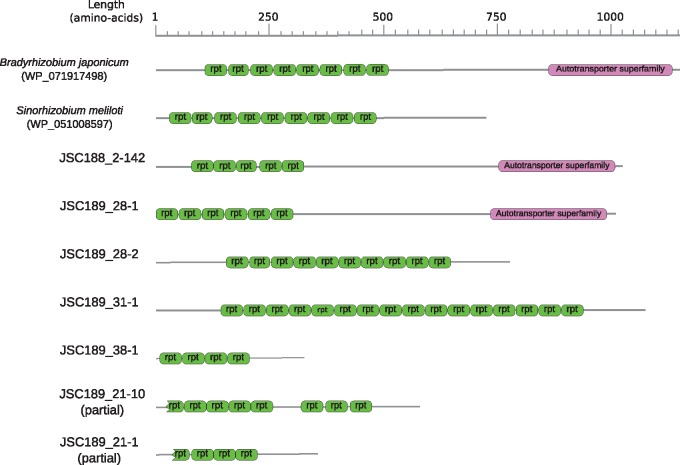
—Autotransporters in the ant-associated *Tokpelaia* species. Domain organization of the inferred autotransporters in the *Dolichoderus* spp. symbionts compared with two reference proteins from the Rhizobiales. Green boxes indicate repeated protein domains.

However, we identified a novel type of autotransporter (of the type V secretion system) in the JSC188 and JSC189 genomes (fig. 7; supplementary table S11, Supplementary Material online). The predicted proteins have variable sizes, ranging from 326 to 1,066 amino acids, and are composed of tandemly repeated domains of 50 amino acids. The N-terminal passenger domain, which is normally translocated across the membrane and presented on the cell surface, was present in all proteins. Two proteins in the family (JSC188_2-141 and JSC189_29-1) also contained a C-terminal outer membrane protein domain. No homologs of this type of autotransporter could be identified in the classical *Bartonella* species, nor in *Ca*. T. hoelldobblerii or in the JSC085 and JSC161 genomes. Proteins containing these domains are present in distantly related α-proteobacteria and γ-proteobacteria (supplementary table S11, Supplementary Material online), but their function has not been studied and the significance of this finding thus remains unknown.

## Discussion

Cook and Davidson (2006) have wondered how herbivorous ants in the genus *Dolichoderus* avoid nutrient imbalance despite their nitrogen-poor diet. The results presented in this paper suggest that Bartonellaceae colonizing their midgut walls are at least a part of the answer.

From the herbivorous host’s perspective, a key benefit of hosting symbionts is the ability to acquire essential amino acids, cofactors and vitamins deficient in the host diet (Mankowski and Morrell 2014; Salem et al. 2014; Manzano-Marín et al. 2015; Wilson and Duncan 2015). Our genomic analyses showed that Bartonellaceae inhabiting the *Dolichoderus* ants have the ability to recycle nitrogenous waste and to synthesize all essential and most nonessential amino acids as well as several vitamins. The amino acid biosynthetic profiles of these bacteria are similar to those of *Blochmannia* in ants and *Buchnera* in aphids, both of which are obligate mutualists (Williams and Wernegreen 2015; Russell et al. 2017). Morphological and ecological evidence have shown that the *Dolichoderus* ants host high quantities of bacteria relative to their body mass, similar to *Camponotus* ants that hosts *Blochmannia* endosymbionts (Sanders et al. 2017). Furthermore, the Bartonellaceae symbionts appear to colonize crypts among the midgut epithelial cells and they may also be abundant in the hindgut, resembling the localization of nutritional endosymbionts such as *Blochmannia* within the midgut wall (Sauer et al. 2002). As yet, it is not clear what proportion of bacterial cells is present in the midgut wall as opposed to the hindgut lumen, and what is the extent of overlap at the genotype level between these two body habitats. Nevertheless, the high density of bacterial cells in the midgut wall does suggest the significance of this microbe for the nutrition of *Dolichoderus* ants.

From a carnivorous host’s perspective, a benefit of its gut microbiota might be the ability to degrade proteins from the diet. In long-lived holometabolous insects such as ants, the adult stage has rather modest demands for nitrogen as a macronutrient, and it has been shown that ant workers die young and colonies collapse when fed a high-protein diet (Dussutour and Simpson 2012). Alternatively, the microbiota of these ants may simply be utilizing the rich supply of nutrients in the ant gut, with limited effects on their hosts. In predatory army ants, Bartonellaceae are sparsely distributed, infecting only some specimens in some colonies (Łukasik 2017), suggesting opportunism and perhaps even pathogenicity. However, whether these bacteria are beneficial, neutral, or harmful to carnivorous ants may very well vary among strains.

Importantly, the repertoire of genes for amino acid and vitamin biosynthesis differed markedly in the Bartonellaceae of herbivorous and carnivorous ants in a way that reflected the different diets of their hosts. The most noticeable difference was the identification of genes for the biosynthesis of histidine and the conversion of glutamine to arginine in the gut symbionts of the *Dolichoderus* ants, contrasted with genes for the import of histidine and arginine and their conversion to glutamine in the symbiont of the *Harpegnathos* ants. In other words, the end products were inferred to be the essential amino acids histidine and arginine in the *Dolichoderus* ants when compared with the nonessential amino acid glutamine in the *Harpegnathos* ants.

It is interesting to note that the major difference in gene contents between these gut symbionts reside in the biosynthesis, conversion and import of three amino acids, histidine, arginine and glutamine, which are thought to play a key role in the symbioses of *Buchnera* with aphids. For example, host regulation of the biosynthetic pathways for aromatic amino acids via glutamine and arginine has been observed during late embryonic stage in *Buchnera* of pea aphids (Rabatel et al. 2013). In the aphid, the nuclear encoded glutamine transporter mediates import of glutamine from the hemolymph to the bacteriocytes (Lu et al. 2016). After its import to the bacteriocyte, glutamine is converted to arginine, which is exported into the hemolymph and from there into the developing embryo. It is hypothesized that arginine may act as an inhibitor of the glutamine transporter (Price et al. 2014). Accordingly, when arginine is no longer needed, the concentration of this amino acid in the hemolymph increases, inhibiting the glutamine transporter and thereby reducing the levels of amino acid biosynthesis in the bacteriocytes. A testable hypothesis is that the *Dolichoderus* ant genome encodes a homologous glutamine transporter that likewise may be used to regulate the synthesis of essential amino acids.

In the *Harpegnathos* symbionts, the import and conversion of histidine and arginine will lead to increased levels of glutamine, which can be a major source of energy and controls insect fecundity by activating cell growth via the TOR pathway (Zhai et al. 2015). The symbiont may thereby exert an influence on ant behavior that goes beyond simply recycling superfluous nitrogen in the diet. Irrespectively, the correlation between the biosynthetic capabilities of the symbionts and the ant diets provide strong support for the hypothesis that the Bartonellaceae have adapted to the *Dolichoderus* ants.

Consistent with a host-beneficial role, the *Dolichoderus* spp. symbionts have a more comprehensive set of amino acid biosynthetic genes than other members of the Bartonellaceae. Furthermore, the JSC188 and the JSC189 strains have a coding content of <80% and thus a high fraction of long noncoding DNA stretches and pseudogenes, indicating that they are in the process of shedding genes not essential for life in the sheltered, controlled environment of midgut crypts.

A similarly broad biosynthetic capability was also found in *B. apis*, a related extracellular gut symbiont that infects honeybees (Segers et al. 2017), which feeds on nectar and is thus also adapted to a very carbohydrate-rich diet. These similarities in metabolic capabilities are consistent with the hypothesis that the last common ancestor of *Bartonella* was a gut symbiont of insects that produced its own amino acids and vitamins (Segers et al. 2017). However, the *B. apis* genomes are almost two times as large (2.6–2.9 Mb) as the *Dolichoderus* spp. symbionts, suggesting that the association with the bee is more recent or that the association is less intimate. Indeed, *B. apis* colonizes the gut lumen as opposed to the crypts, and is thus exposed to a much more variable habitat. Differences in the population genetics of transmission as a result of host social biology may also play a role. Colony size has been shown to correlate with gut bacterial diversity in bees (Kwong et al. 2017), and gene loss due to accumulation of nonfatal deleterious alleles should be stronger in symbionts that undergo more severe population bottlenecks (McCutcheon and Moran 2012). Although *Dolichoderus* have large colonies, their bacterial symbionts may experience much more severe bottlenecks during colony reproduction, in which solitary foundress queens disperse; by comparison, honeybee colonies reproduce via colony fission of thousands of individuals.

Of the four *Dolichoderus* spp. symbionts strains analyzed here, the JSC161 strain has the smallest and most gene-dense genome as well as the lowest genomic GC content. It has also accumulated nucleotide substitutions more rapidly than the other symbiont genomes. It thus appears as if the host-adaptation process has been more extensive in this strain. Notably, genes involved in the recombination repair RecFOR pathway were identified among the functions absent from this genome, but present in the other symbiont genomes. It is thus tempting to speculate that reduced levels of homologous recombination in the JSC161 genome may explain the increased rates of nucleotide substitutions and gene loss. Among the 49 genes uniquely present in the JSC161 strain, we identified the *nadABC* genes for the synthesis of vitamin B3, the acquisition of which may also have played a role the process of host-adaptation. In this context, it is interesting to note that the horizontal acquisition of an operon for biotin biosynthesis (vitamin B7) has transformed *Wolbachia* of the bedbug *Cimex lecturalius* into a mutualist (Nikoh et al. 2014; Gerth and Bleidorn 2017).

Finally, we may wonder whether the bacterial infections have had an impact on the lifestyle and ecology of the ants. We are becoming increasingly aware of the broad distribution of endosymbionts and gut microbes closely associated with ants and other insect hosts. It seems likely that the provisioning of essential amino acids from the gut microbiome has triggered the success and spread of the *Dolichoderus* lineage, just like the acquisition of *Blochmannia*, enabled the evolutionary success of primarily herbivorous ants of the genus *Camponotus* (Davidson et al. 2003; Cook and Davidson 2006; Feldhaar et al. 2007). Such a scenario assumes that species from outside of Peruvian Amazon also contain the same type of gut microbes, that the infection was established at an early stage of the diversification of this group and that the hosts and the symbionts have codiversified. More detailed phylogenetic and population-wide studies are needed to fully understand the role that these bacteria have played in the biology of the ants.

We conclude that the adaptation to midgut crypt environment of herbivorous *Dolichoderus* ants has been associated with reductive evolution of the *Tokpelaia* symbionts’ genomes, whereas the nutritional content of the ant’s diets (carbohydrate vs. proteins) have influenced the metabolic capabilities of these bacteria. The striking complementarity between the metabolic profile of the symbionts and the diet of the hosts indicates a stable, long-term, mutually beneficial association between ants and their gut bacteria. By synthesizing essential nutrients, the symbionts have likely impacted the evolution and ecology of *Dolichoderus* spp., allowing them to colonize novel niches where these compounds cannot easily be obtained from the available foods. Future, broad surveys will clarify to what extent microbial associations have promoted ant diversity in these and other habitats, and which microbes are the most prolific contributors.

## Supplementary Material

Supplementary data are available at *Genome Biology and Evolution* online.

## Supplementary Material

Supplementary Data
