# Supplementary material for: Genome Evolution of Bartonellaceae Symbionts of Ants at the Opposite Ends of the Trophic Scale
Source: Genome Biol Evol. 2018 Jul 2;10(7):1687–704. doi: 10.1093/gbe/evy126 (PMC6044324; doi:10.1093/gbe/evy126)
Supplement: Supplementary Data [file evy126_suppl.zip › SI.pdf]

Supporting information

Genome evolution of Bartonellaceae symbionts of ants at the opposite ends of the trophic scale

Running title: Genomics of Bartonellaceae in ants

Gaelle Bisch[1], Minna-Maria Neuvonen[1], Naomi E. Pierce[2], Jacob A. Russell[3], Ryuichi Koga[4], Jon G. Sanders[2,5], Piotr Łukasik[3,6], and Siv G. E. Andersson[1*]

Supplementary figures

Supplementary fig. S1. Normalized bacterial abundance in the guts of Peruvian ants. Data have been taken from (Sanders *et al*., 2017) and show 16S rRNA qPCR counts, minus mean non-template control counts, divided by total DNA concentration as an internal proxy for body size. Each data point represents an individual, grouped by all arboreal nesting ants, all ground nesting ants, and each of the three *Dolichoderus* colonies analyzed in this paper for which qPCR data were available.

Supplementary fig. S2. Relative abundance of bacterial taxa in the *Dolichoderus* ants. **(A)** The relative abundance of bacterial 97% OTUs in surface-sterilized gasters of eleven workers, each from a different colony of *Dolichoderus* sp. from the Peruvian Amazon, based on 16S rRNA amplicon sequencing. Relationships among OTUs are demonstrated using a maximum likelihood phylogeny for the most abundant genotypes from each OTU. **(B)** The relative abundance of unique genotypes from four Bartonellaceae OTUs. Only unique genotypes identified as error-free (i.e., which made up not less than 5% of the OTU in at least one ant, and which differed from considerably more abundant genotypes in the same ant at more than one nucleotide position) were used for comparisons. The relationships among unique genotypes are illustrated using a maximum likelihood tree. Check marks indicate unique genotypes that perfectly matched 16S rRNA sequences amplified and Sanger-sequenced using universal eubacterial primers from dissected midgut walls of other workers from the same ant colonies. In workers from colony JSC161, Sanger traces were polymorphic at the single position that differed between the genotypes shown here. In panels A and B, asterisks indicate colonies for which metagenomic sequencing of DNA extracted from midgut walls was performed, and triangle indicates colony that was used for microscopy.

Supplementary fig. S3. Taxon-annotated GC-coverage plots for four *Dolichoderus* midgut wall metagenomes reveal that Bartonellaceae are the dominant microbes in these host tissues. In each metagenome, all assembled scaffolds with high similarity (E < e-50) to bacterial genomic references, as well as the 10,000 longest scaffolds from among those remaining, were plotted based on their % GC content (x-axis) and their depth of sequencing coverage (y-axis, log scale). Circle size represents the length of the scaffold, and the color corresponds to the taxonomic assignments of the contigs. Contigs with the highest sequence similarity to Bartonellaceae are shown in pink.

Supplementary fig. S4. Lack of conservation of gene order. Comparison of gene order in the genome contigs of the *Dolichoderus* spp. symbionts and the genome of *Ca*. T. hoelldobblerii. Each dot represents a match between Promer-predicted proteins. Forward matches are colored in red and reverse matches in blue. Gene location is indicated for *Ca*. T. hoelldobblerii on the x-axis and the contig number is shown for the *Dolichoderus* spp. symbionts on the y-axis.

Supplementary fig. S5. Box plot of GC3% for each contig in the *Dolichoderus* spp. symbionts genomes. Bar indicates the limit with contigs shorter than 20 000 bp.

Supplementary fig. S6. Frequency of intergenic regions of different sizes in the

Dolichoderus spp. symbionts genomes.

Supplementary fig. S7. GC content and length of intergenic regions in the *Dolichoderus* spp. symbionts genomes. Red dots indicate hits in BLAST searches ($E < 10^{-10}$). Red line indicates the mean GC% for the strain.

Supplementary fig. S8. Rapid rates of sequence evolution in the JSC161 strain. The distribution of the relative branch length for the JSC161 strain in all single protein trees is shown. Phylogenetic trees were inferred for each single-copy ortholog, and the ratio between the length of the branch to the JSC161 branch and the average length of the three other *Dolichoderus* spp. symbionts branches was plotted.

Supplementary fig. S9 Gene flux analysis. Genes families gains and losses inferred with **(A)** individual *Dolichoderus* spp. symbionts genomes and **(B)** combined *Dolichoderus* spp. symbionts genomes. Gains of protein families are shown in green, while losses are shown in red. Total number of ancestral protein families is indicated in black.

Supplementary fig. S10. Phylogeny of proteins involved in the degradation of urea. Phylogenetic trees were inferred based on **(A)** protein in the urease complex (UreC) and **(B)** the glutamine synthetase (GlnA). Ant symbionts are shown in red colour; *Bartonella* species in blue, and outgroups in gray. Only bootstrap values >80% are shown.

Supplementary fig. S11. Phylogeny of proteins involved in the synthesis of pyridoxine. Phylogenetic trees were inferred based on **(A)** PdxJ **(B)** PdxA and **(C)** PdxK. Ant symbionts are shown in red colour; *Bartonella* species in blue, and outgroups in gray. Only bootstrap values >80% are shown.

Supplementary Tables

Supplementary table S1. Primers and probes used in this study.

Supplementary table S2. Contig information. Length, codon adaptation index (CAI), GC3%, GC% and Blast hits (which BLAST, which e-value) for each contig in the assembled sequence data from the *Dolichoderus* spp. symbionts.

Supplementary table S3. Genomes used in this study.

Supplementary table S4. General characteristics of the *Dolichoderus* spp. symbionts genomes assemblies. Completeness was estimated using the method of Land *et al.* (2014).

Supplementary table S5. BLASTx hits (e-value 10^{-10}) for intergenic regions.

Supplementary table S6. List of proteins used in the concatenated protein tree.

Supplementary table S7. 16S rRNA identities and the average amino acid identity (AAI) values for the *Dolichoderus* spp. symbionts and *Ca*. Tokpelaia hoelldobblerii. The first number is the 16S rRNA identity value, the second one the AAI value. All numbers are given in percentages.

Supplementary tables S8. Shared and specific protein families for *Ca*. T. hoelldobblerii and the four *Dolichoderus* spp. strains. See tabs for content.

- **(a)** *Dolichoderus* sp. symbiont JSC085 specific protein families.
- **(b)** *Dolichoderus* sp. symbiont JSC161 specific protein families.
- **(c)** *Dolichoderus* sp. symbiont JSC188 specific protein families.
- **(d)** *Dolichoderus* sp. symbiont JSC189 specific protein families.
- **(e)** *Ca*. Tokpelaia hoelldobblerii specific protein families.
- **(f)** Protein families shared by the four *Dolichoderus* spp. symbiont
- **(g)** Protein families in the core genome of the genus *Tokpelaia*.

Supplementary tables S9. Protein family gains and losses in *Ca*. T. hoelldobblerii, the four combined *Dolichoderus* spp. symbionts ancestor and the common ancestor of the *Tokpelaia* sp. See tabs for content.

- (a) Protein families gained in the *Dolichoderus* spp. symbionts.
- (b) Protein families lost in the *Dolichoderus* spp. symbionts.
- (c) Protein families gained in *Ca*. Tokpelaia hoelldobblerii.
- (d) Protein families lost in *Ca*. Tokpelaia hoelldobblerii.
- (e) Protein families lost in the genus *Bartonella*.
- (f) Protein families lost in the genus *Tokpelaia*.
- (g) Protein families lost in the Dolichoderus spp. symbiont JSC161.

Supplementary table S10. Comparison of the number of genes coding for pathways involved in the biosynthesis of amino acids and vitamins in the *Dolichoderus* spp. symbionts, *Ca*. T. hoelldobblerii and other endosymbionts.

Supplementary table S11. Lipopolysaccharide and peptidoglycan synthesis genes.

Supplementary table S12. Domains **(A)** and blastP hits **(B)** for the autotransporters in the strains JSC188 and JSC189.

Fig. S1

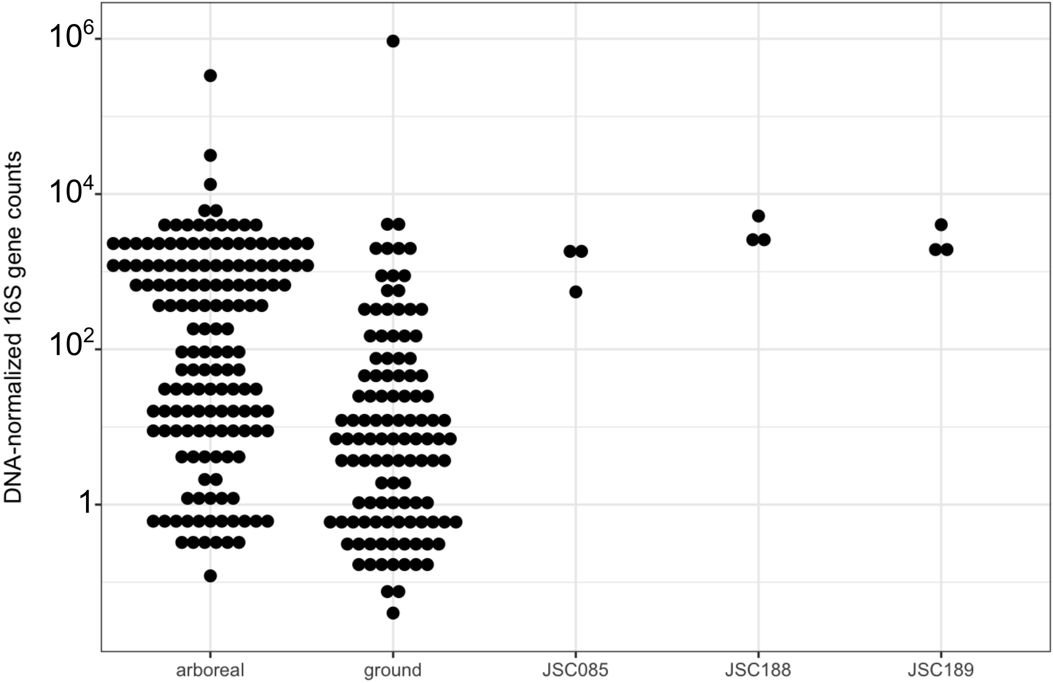
DNA-normalized 16S gene counts
10^6
10^4
10^2
1
arboreal
ground
JSC085
JSC188
JSC189

Fig. S2

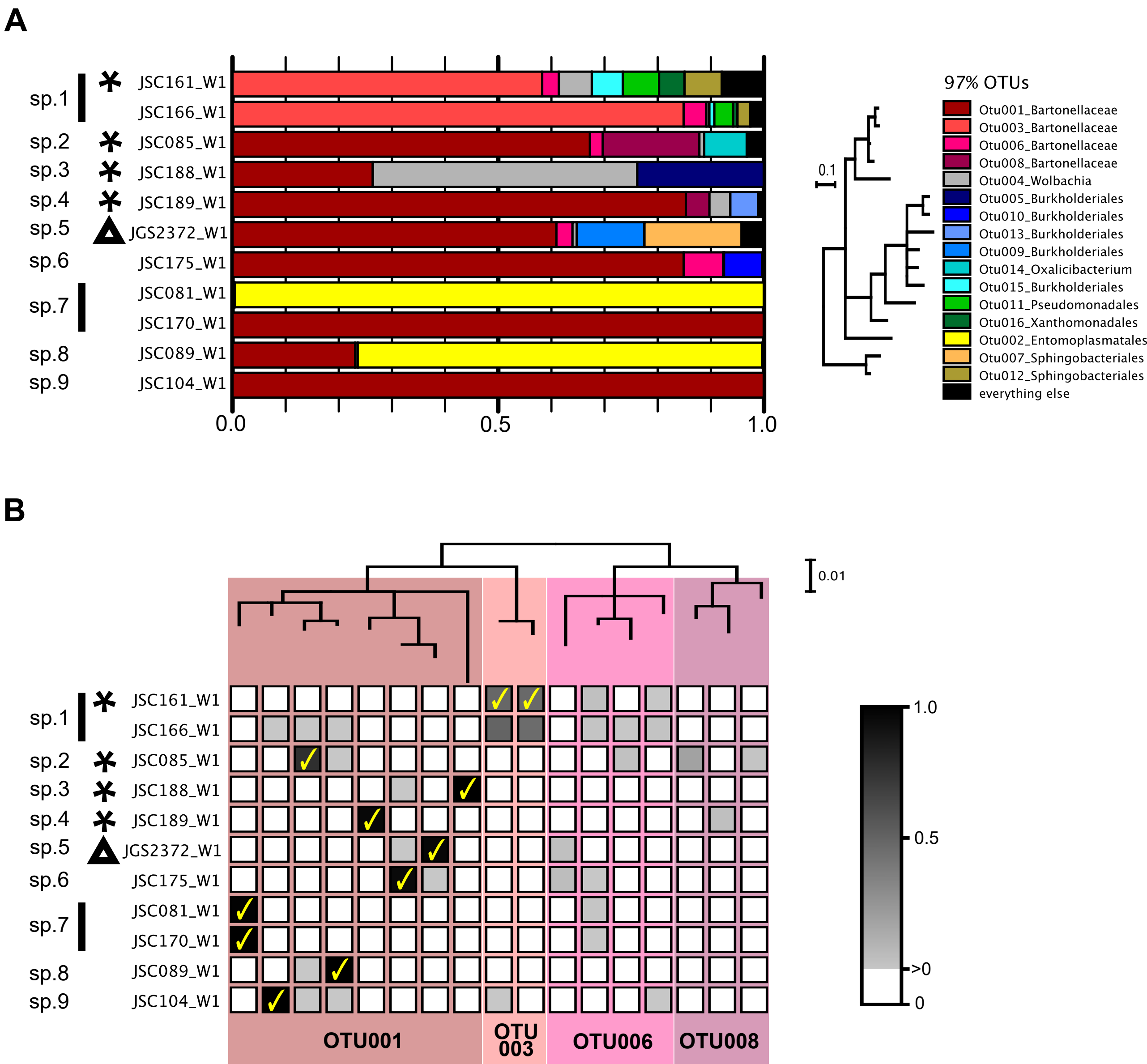

A
97% OTUs
Otu001_Bartonellaceae
Otu003_Bartonellaceae
Otu006_Bartonellaceae
Otu008_Bartonellaceae
Otu004_Wolbachia
Otu005_Burkholderiales
Otu010_Burkholderiales
Otu013_Burkholderiales
Otu009_Burkholderiales
Otu014_Oxalicibacterium
Otu015_Burkholderiales
Otu011_Pseudomonadales
Otu016_Xanthomonadales
Otu002_Entomoplasmatales
Otu007_Sphingobacteriales
Otu012_Sphingobacteriales
everything else
0.1
sp.1 JSC161_W1
JSC166_W1
sp.2 JSC085_W1
sp.3 JSC188_W1
sp.4 JSC189_W1
sp.5 JGS2372_W1
sp.6 JSC175_W1
sp.7 JSC081_W1
JSC170_W1
sp.8 JSC089_W1
sp.9 JSC104_W1
0.0
0.5
1.0
B
0.01
1.0
0.5
>0
0
OTU001
OTU 003
OTU006
OTU008

Fig. S3

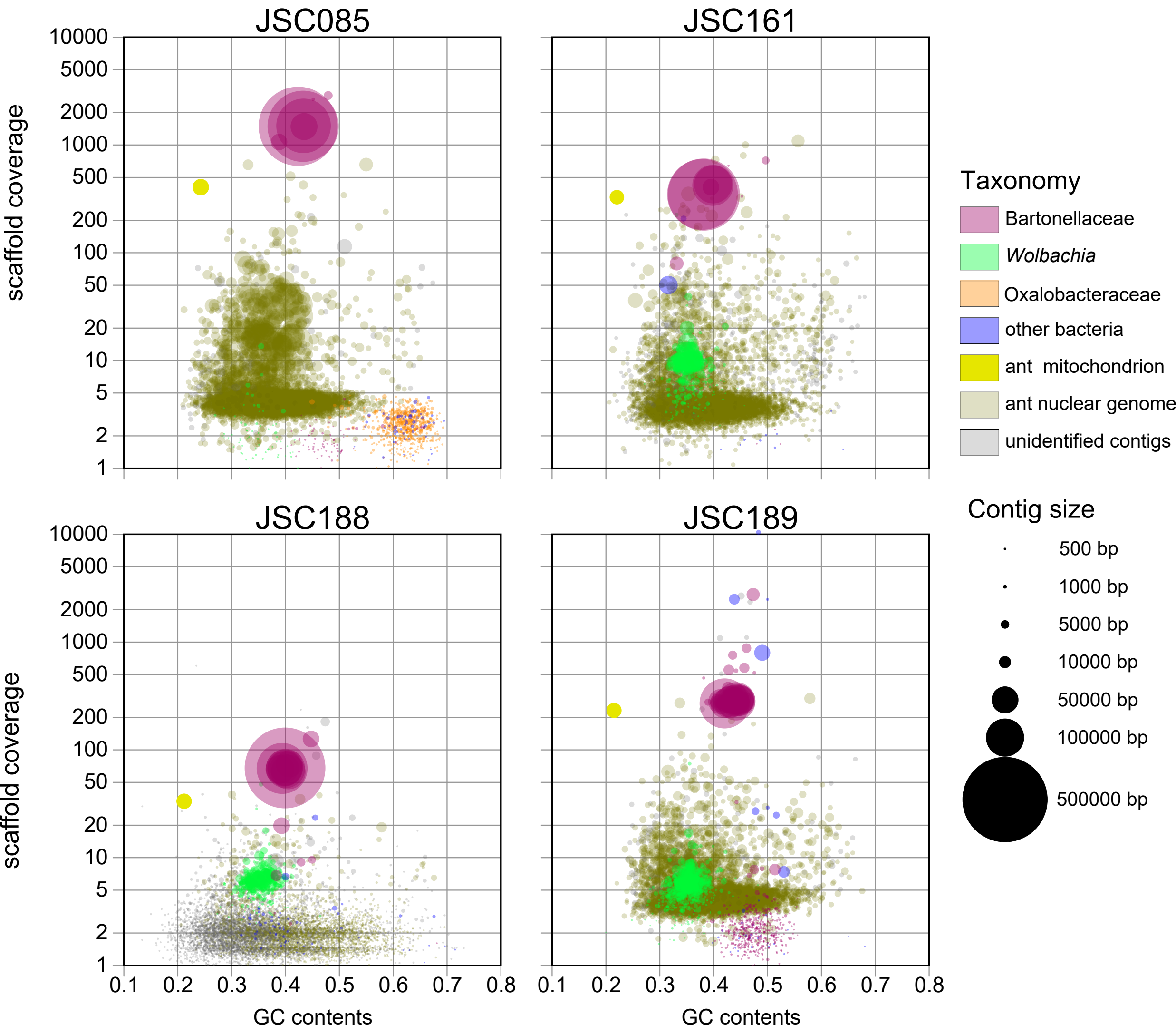

JSC085
JSC161
JSC188
JSC189
scaffold coverage
10000
5000
2000
1000
500
200
100
50
20
10
5
2
1
GC contents
0.1
0.2
0.3
0.4
0.5
0.6
0.7
0.8
Taxonomy
Bartonellaceae
Wolbachia
Oxalobacteraceae
other bacteria
ant mitochondrion
ant nuclear genome
unidentified contigs
Contig size
500 bp
1000 bp
5000 bp
10000 bp
50000 bp
100000 bp
500000 bp

Fig. S4

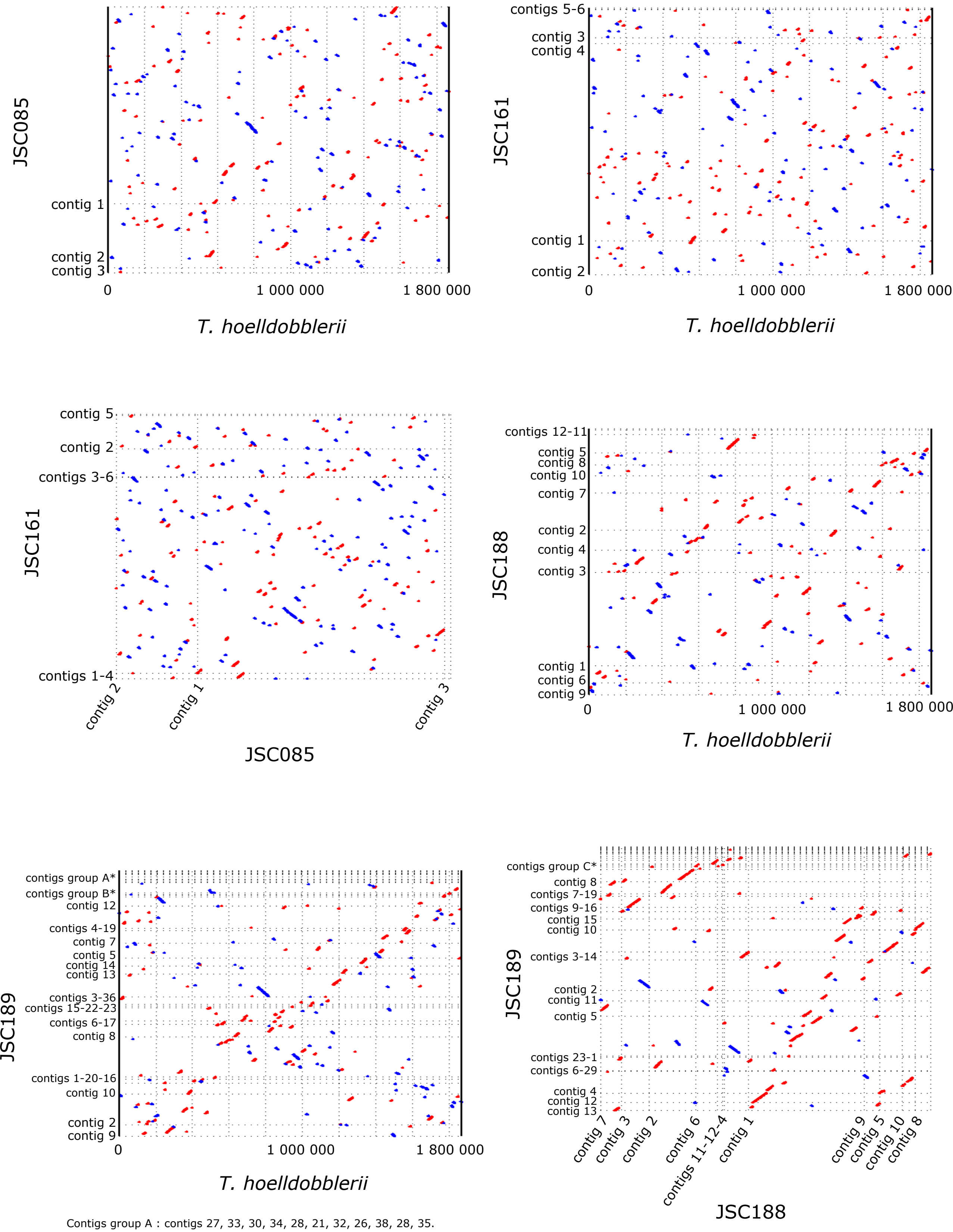

Contigs group A : contigs 27, 33, 30, 34, 28, 21, 32, 26, 38, 28, 35.
Contigs group B : contigs 11, 24, 29, 25.

Contigs group C : contigs 37, 39, 31, 33, 30, 36, 34, 26, 38, 28, 35, 25, 24, 20, 22, 17, 27, 21, 18.

Fig. S5

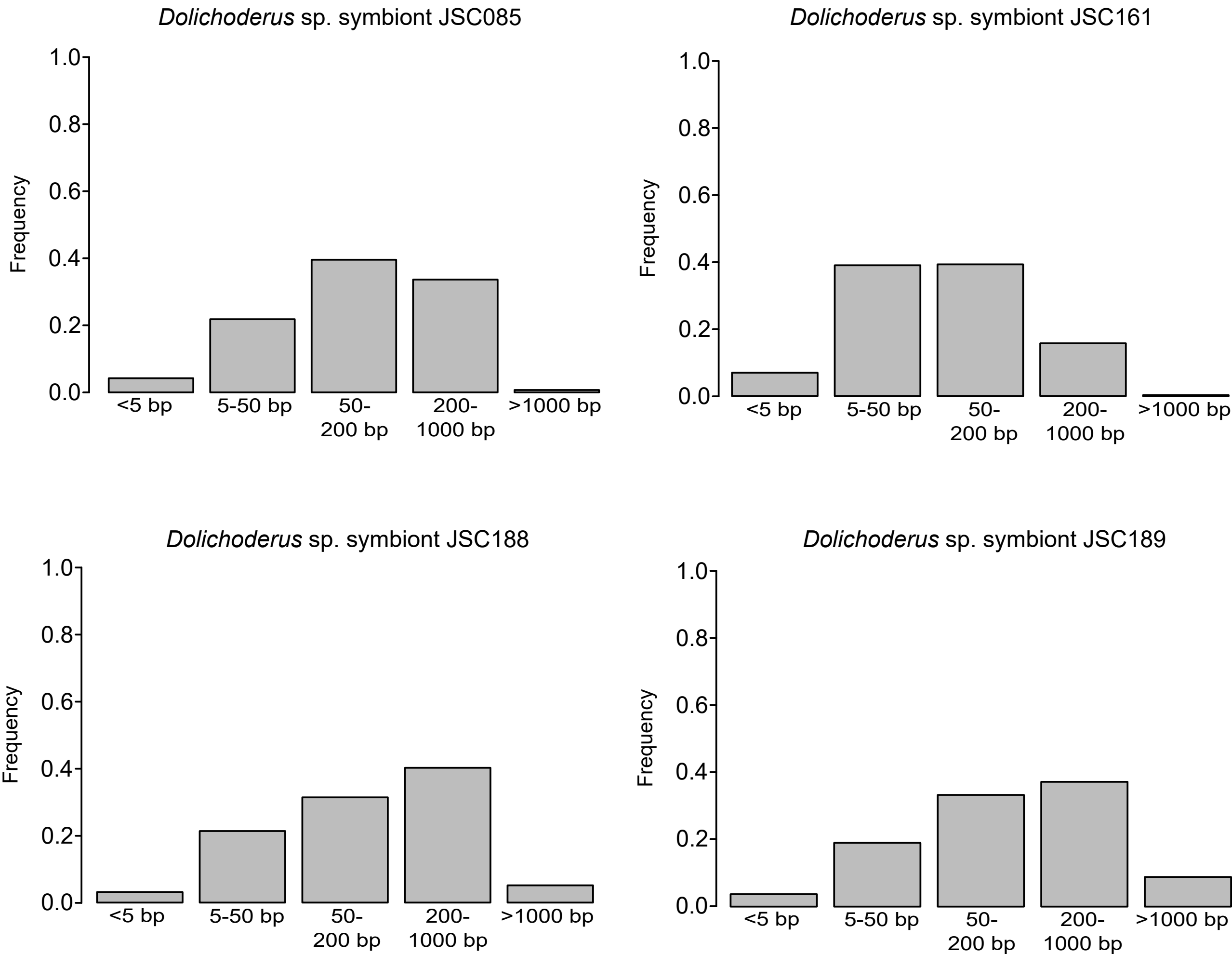
Dolichoderus sp. symbiont JSC085
Dolichoderus sp. symbiont JSC161
Dolichoderus sp. symbiont JSC188
Dolichoderus sp. symbiont JSC189
Frequency
1.0
0.8
0.6
0.4
0.2
0.0
<5 bp
5-50 bp
50-
200 bp
200-
1000 bp
>1000 bp

Fig. S6

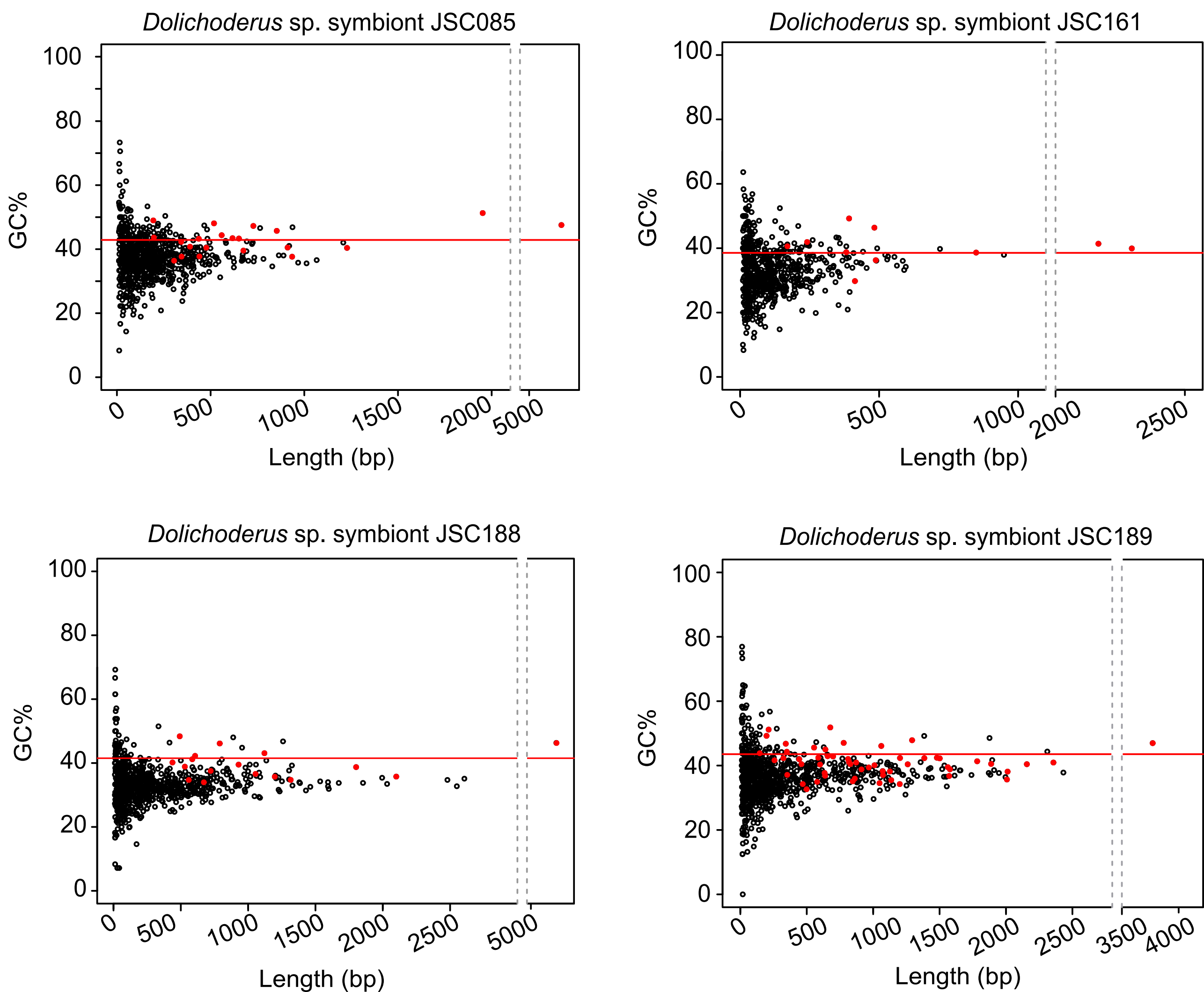

Dolichoderus sp. symbiont JSC085
Dolichoderus sp. symbiont JSC161
Dolichoderus sp. symbiont JSC188
Dolichoderus sp. symbiont JSC189
GC%
Length (bp)
0
20
40
60
80
100
0
500
1000
1500
2000
2500
3500
4000
5000

Fig. S7

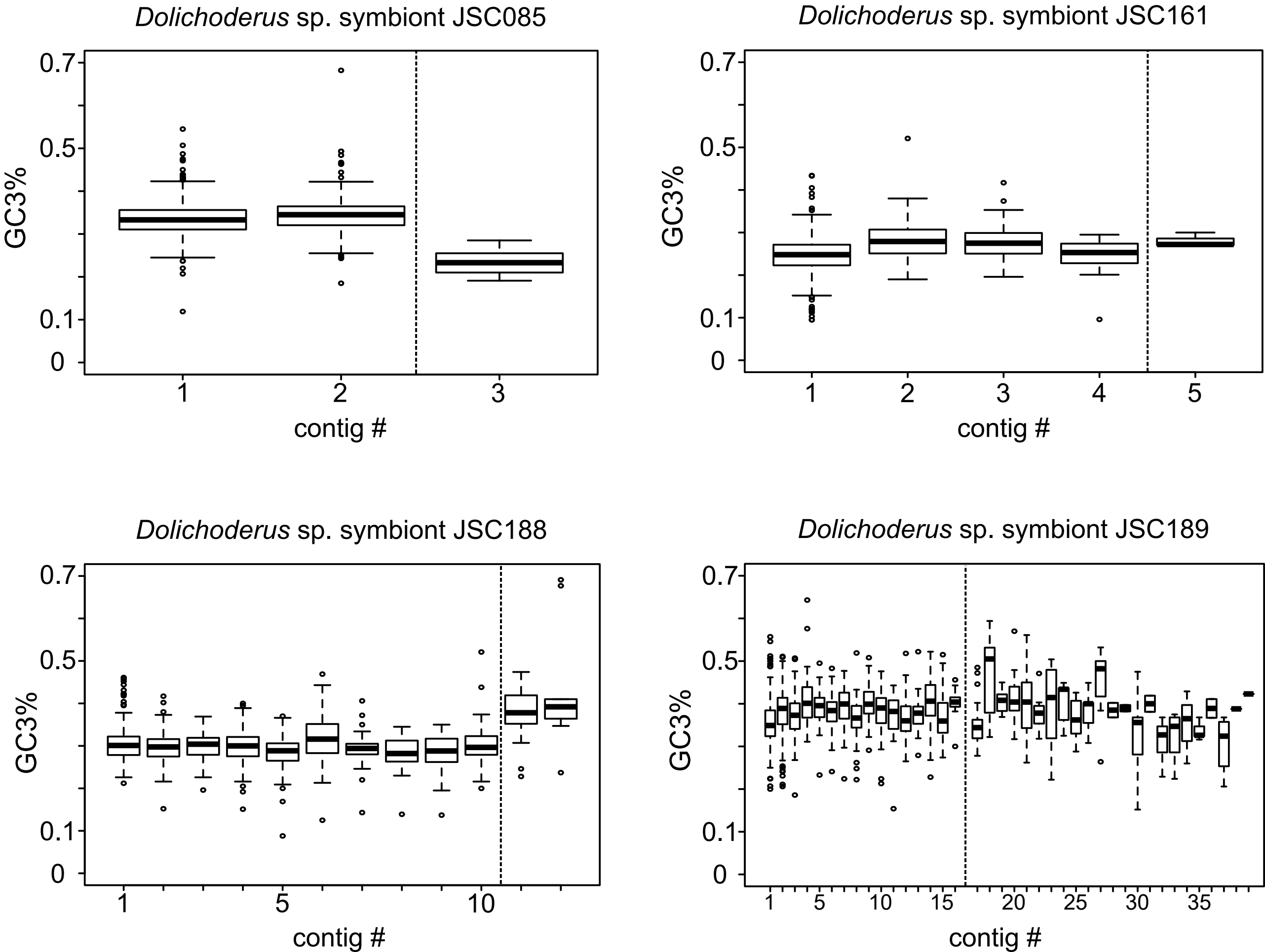
Dolichoderus sp. symbiont JSC085
Dolichoderus sp. symbiont JSC161
Dolichoderus sp. symbiont JSC188
Dolichoderus sp. symbiont JSC189
GC3%
contig #
0
0.1
0.5
0.7
1
2
3
4
5
10
15
20
25
30
35

Fig. S8

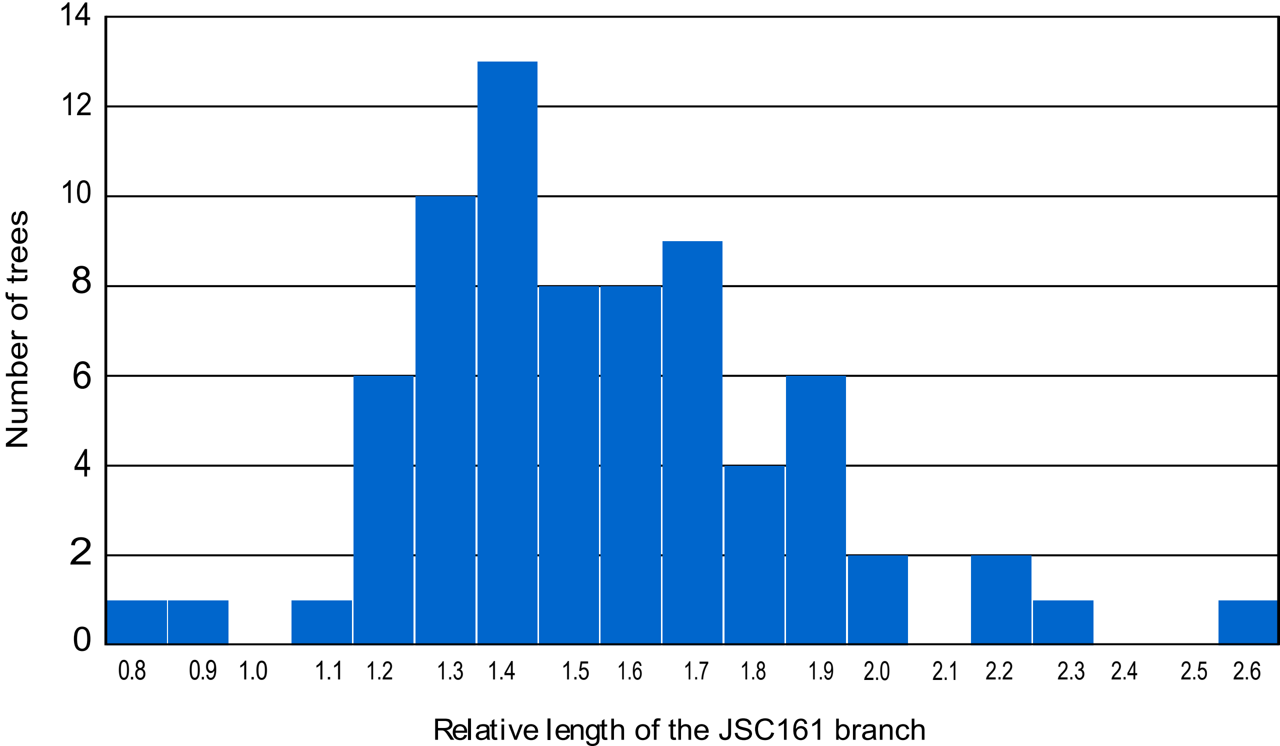

Number of trees
14
12
10
8
6
4
2
0
0.8
0.9
1.0
1.1
1.2
1.3
1.4
1.5
1.6
1.7
1.8
1.9
2.0
2.1
2.2
2.3
2.4
2.5
2.6
Relative length of the JSC161 branch

Fig. S9

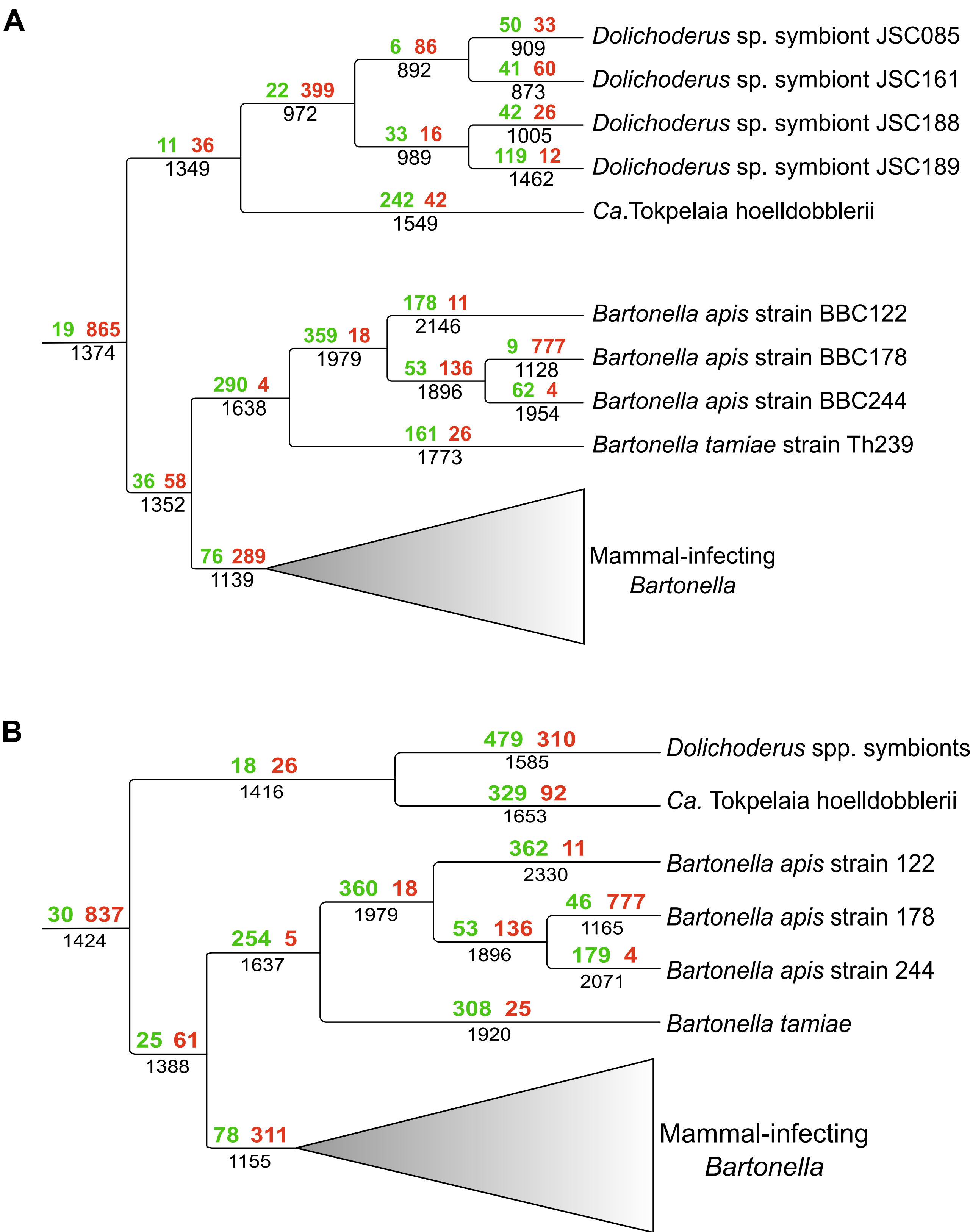
A
50 33
909
Dolichoderus sp. symbiont JSC085
41 60
873
Dolichoderus sp. symbiont JSC161
6 86
892
22 399
972
42 26
1005
Dolichoderus sp. symbiont JSC188
33 16
989
119 12
1462
Dolichoderus sp. symbiont JSC189
11 36
1349
242 42
1549
Ca.Tokpelaia hoelldobblerii
178 11
2146
Bartonella apis strain BBC122
19 865
1374
359 18
1979
9 777
1128
Bartonella apis strain BBC178
53 136
1896
62 4
1954
Bartonella apis strain BBC244
290 4
1638
161 26
1773
Bartonella tamiae strain Th239
36 58
1352
76 289
1139
Mammal-infecting Bartonella
B
479 310
1585
Dolichoderus spp. symbionts
18 26
1416
329 92
1653
Ca. Tokpelaia hoelldobblerii
362 11
2330
Bartonella apis strain 122
360 18
1979
30 837
1424
46 777
1165
Bartonella apis strain 178
53 136
1896
254 5
1637
179 4
2071
Bartonella apis strain 244
308 25
1920
Bartonella tamiae
25 61
1388
78 311
1155
Mammal-infecting Bartonella

Fig. S10

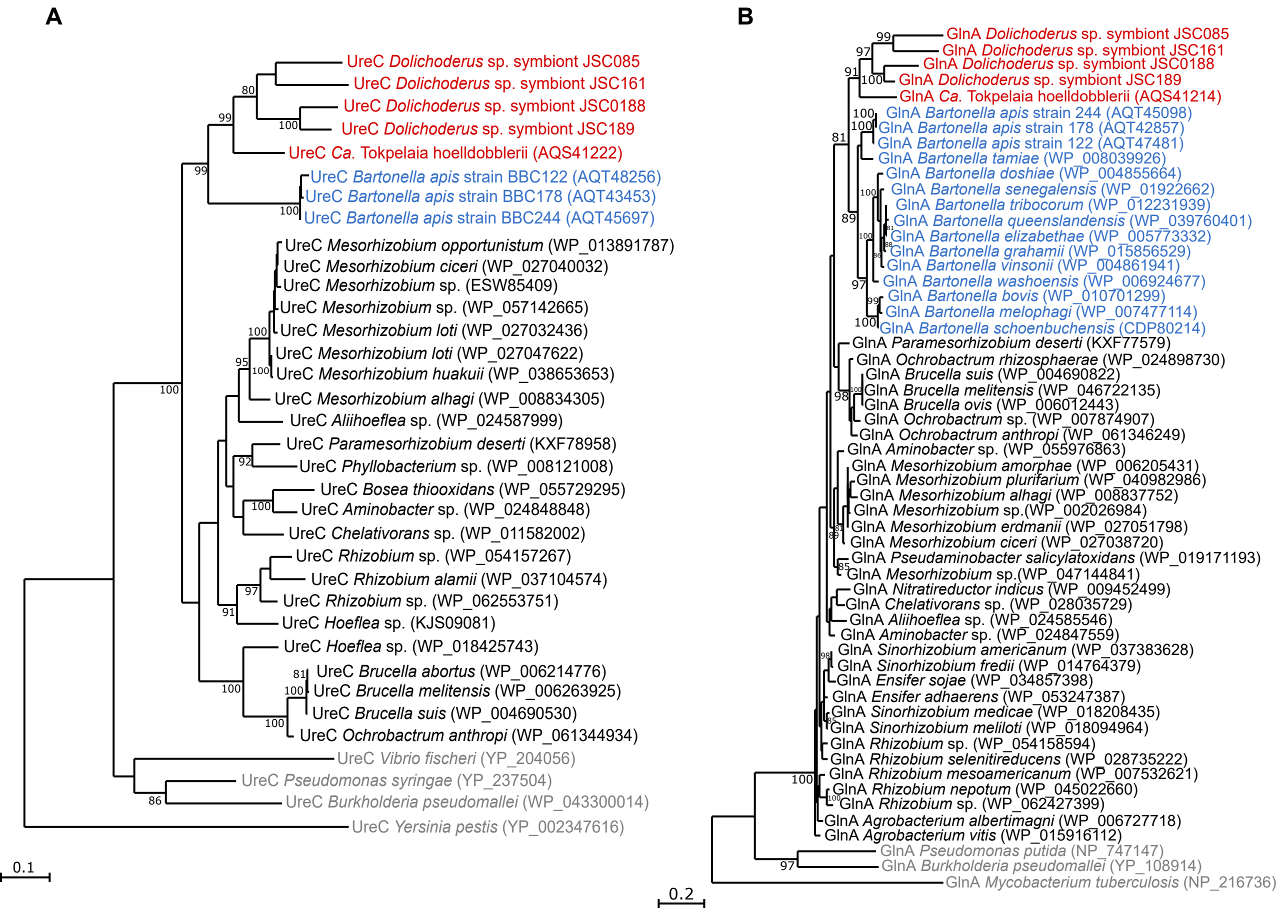
A
UreC Dolichoderus sp. symbiont JSC085
UreC Dolichoderus sp. symbiont JSC161
UreC Dolichoderus sp. symbiont JSC0188
UreC Dolichoderus sp. symbiont JSC189
UreC Ca. Tokpelaia hoelldobblerii (AQS41222)
UreC Bartonella apis strain BBC122 (AQT48256)
UreC Bartonella apis strain BBC178 (AQT43453)
UreC Bartonella apis strain BBC244 (AQT45697)
UreC Mesorhizobium opportunistum (WP_013891787)
UreC Mesorhizobium ciceri (WP_027040032)
UreC Mesorhizobium sp. (ESW85409)
UreC Mesorhizobium sp. (WP_057142665)
UreC Mesorhizobium loti (WP_027032436)
UreC Mesorhizobium loti (WP_027047622)
UreC Mesorhizobium huakuii (WP_038653653)
UreC Mesorhizobium alhagi (WP_008834305)
UreC Aliihoeflea sp. (WP_024587999)
UreC Paramesorhizobium deserti (KXF78958)
UreC Phyllobacterium sp. (WP_008121008)
UreC Bosea thiooxidans (WP_055729295)
UreC Aminobacter sp. (WP_024848848)
UreC Chelativorans sp. (WP_011582002)
UreC Rhizobium sp. (WP_054157267)
UreC Rhizobium alamii (WP_037104574)
UreC Rhizobium sp. (WP_062553751)
UreC Hoeflea sp. (KJS09081)
UreC Hoeflea sp. (WP_018425743)
UreC Brucella abortus (WP_006214776)
UreC Brucella melitensis (WP_006263925)
UreC Brucella suis (WP_004690530)
UreC Ochrobactrum anthropi (WP_061344934)
UreC Vibrio fischeri (YP_204056)
UreC Pseudomonas syringae (YP_237504)
UreC Burkholderia pseudomallei (WP_043300014)
UreC Yersinia pestis (YP_002347616)
0.1
B
GlnA Dolichoderus sp. symbiont JSC085
GlnA Dolichoderus sp. symbiont JSC161
GlnA Dolichoderus sp. symbiont JSC0188
GlnA Dolichoderus sp. symbiont JSC189
GlnA Ca. Tokpelaia hoelldobblerii (AQS41214)
GlnA Bartonella apis strain 244 (AQT45098)
GlnA Bartonella apis strain 178 (AQT42857)
GlnA Bartonella apis strain 122 (AQT47481)
GlnA Bartonella tamiae (WP_008039926)
GlnA Bartonella doshiae (WP_004855664)
GlnA Bartonella senegalensis (WP_01922662)
GlnA Bartonella tribocorum (WP_012231939)
GlnA Bartonella queenslandensis (WP_039760401)
GlnA Bartonella elizabethae (WP_005773332)
GlnA Bartonella grahamii (WP_015856529)
GlnA Bartonella vinsonii (WP_004861941)
GlnA Bartonella washoensis (WP_006924677)
GlnA Bartonella bovis (WP_010701299)
GlnA Bartonella melophagi (WP_007477114)
GlnA Bartonella schoenbuchensis (CDP80214)
GlnA Paramesorhizobium deserti (KXF77579)
GlnA Ochrobactrum rhizosphaerae (WP_024898730)
GlnA Brucella suis (WP_004690822)
GlnA Brucella melitensis (WP_046722135)
GlnA Brucella ovis (WP_006012443)
GlnA Ochrobactrum sp. (WP_007874907)
GlnA Ochrobactrum anthropi (WP_061346249)
GlnA Aminobacter sp. (WP_055976863)
GlnA Mesorhizobium amorphae (WP_006205431)
GlnA Mesorhizobium plurifarium (WP_040982986)
GlnA Mesorhizobium alhagi (WP_008837752)
GlnA Mesorhizobium sp.(WP_002026984)
GlnA Mesorhizobium erdmanii (WP_027051798)
GlnA Mesorhizobium ciceri (WP_027038720)
GlnA Pseudaminobacter salicylatoxidans (WP_019171193)
GlnA Mesorhizobium sp.(WP_047144841)
GlnA Nitratireductor indicus (WP_009452499)
GlnA Chelativorans sp. (WP_028035729)
GlnA Aliihoeflea sp. (WP_024585546)
GlnA Aminobacter sp. (WP_024847559)
GlnA Sinorhizobium americanum (WP_037383628)
GlnA Sinorhizobium fredii (WP_014764379)
GlnA Ensifer sojae (WP_034857398)
GlnA Ensifer adhaerens (WP_053247387)
GlnA Sinorhizobium medicae (WP_018208435)
GlnA Sinorhizobium meliloti (WP_018094964)
GlnA Rhizobium sp. (WP_054158594)
GlnA Rhizobium selenitireducens (WP_028735222)
GlnA Rhizobium mesoamericanum (WP_007532621)
GlnA Rhizobium nepotum (WP_045022660)
GlnA Rhizobium sp. (WP_062427399)
GlnA Agrobacterium albertimagni (WP_006727718)
GlnA Agrobacterium vitis (WP_015916112)
GlnA Pseudomonas putida (NP_747147)
GlnA Burkholderia pseudomallei (YP_108914)
GlnA Mycobacterium tuberculosis (NP_216736)
0.2

Fig. S11

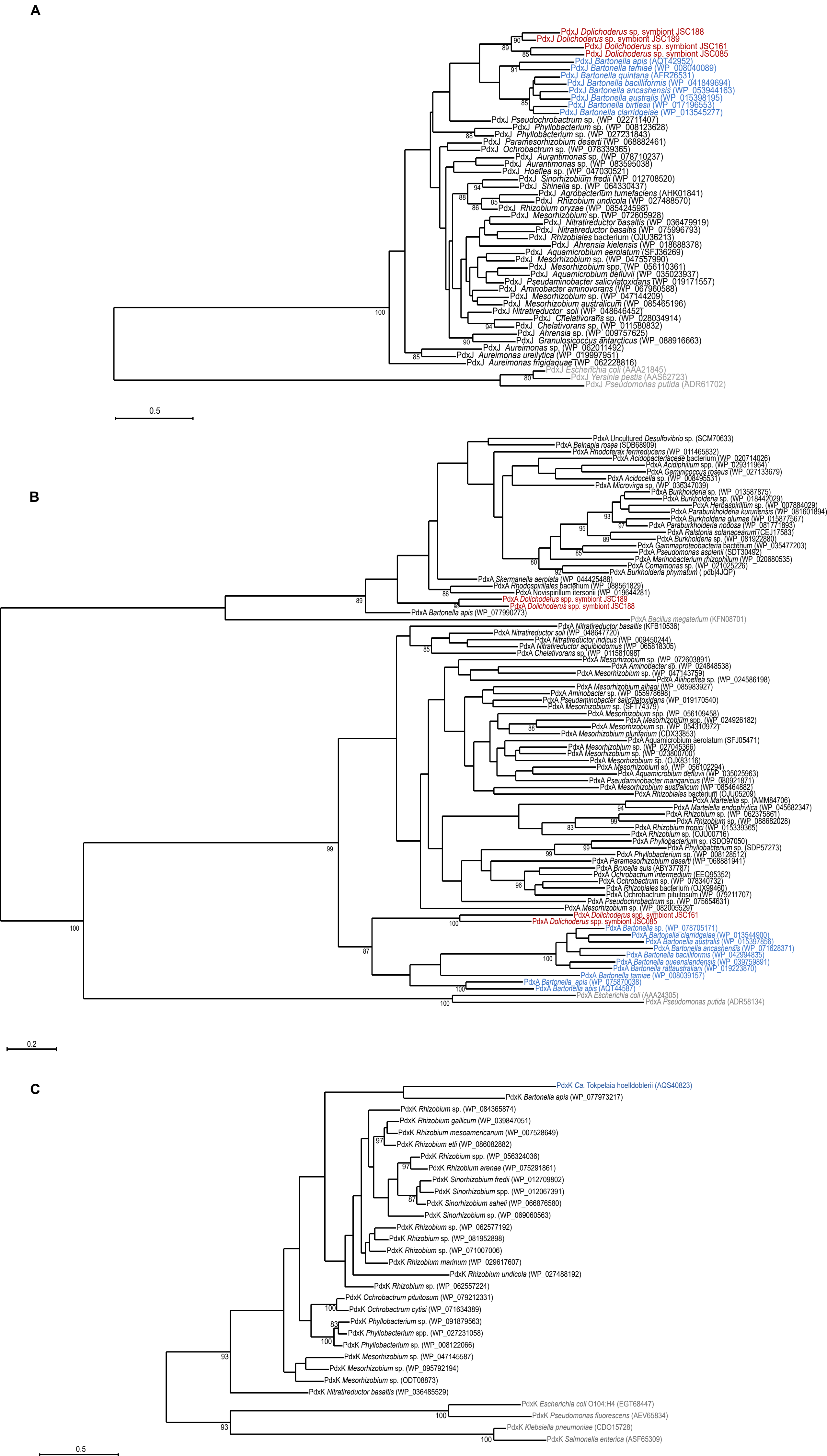
A
PdxJ Dolichoderus sp. symbiont JSC188
PdxJ Dolichoderus sp. symbiont JSC189
PdxJ Dolichoderus sp. symbiont JSC161
PdxJ Dolichoderus sp. symbiont JSC085
PdxJ Bartonella apis (AQT42952)
PdxJ Bartonella tamiae (WP_008040089)
PdxJ Bartonella quintana (AFR26531)
PdxJ Bartonella bacilliformis (WP_041849694)
PdxJ Bartonella ancashensis (WP_053944163)
PdxJ Bartonella australis (WP_015398195)
PdxJ Bartonella birtlesii (WP_017196553)
PdxJ Bartonella clarridgeiae (WP_013545277)
PdxJ Pseudochrobactrum sp. (WP_022711407)
PdxJ Phyllobacterium sp. (WP_008123628)
PdxJ Phyllobacterium sp. (WP_027231843)
PdxJ Paramesorhizobium deserti (WP_068882461)
PdxJ Ochrobactrum sp. (WP_078339365)
PdxJ Aurantimonas sp. (WP_078710237)
PdxJ Aurantimonas sp. (WP_083595038)
PdxJ Hoeflea sp. (WP_047030521)
PdxJ Sinorhizobium fredii (WP_012708520)
PdxJ Shinella sp. (WP_064330437)
PdxJ Agrobacterium tumefaciens (AHK01841)
PdxJ Rhizobium undicola (WP_027488570)
PdxJ Rhizobium oryzae (WP_085424598)
PdxJ Mesorhizobium sp. (WP_072605928)
PdxJ Nitratireductor basaltis (WP_036479919)
PdxJ Nitratireductor basaltis (WP_075996793)
PdxJ Rhizobiales bacterium (OJU36213)
PdxJ Ahrensia kielensis (WP_018688378)
PdxJ Aquamicrobium aerolatum (SFJ36269)
PdxJ Mesorhizobium sp. (WP_047557990)
PdxJ Mesorhizobium spp. (WP_056110361)
PdxJ Aquamicrobium defluvii (WP_035023937)
PdxJ Pseudaminobacter salicylatoxidans (WP_019171557)
PdxJ Aminobacter aminovorans (WP_067960588)
PdxJ Mesorhizobium sp. (WP_047144209)
PdxJ Mesorhizobium australicum (WP_085465196)
PdxJ Nitratireductor soli (WP_048646452)
PdxJ Chelativorans sp. (WP_028034914)
PdxJ Chelativorans sp. (WP_011580832)
PdxJ Ahrensia sp. (WP_009757625)
PdxJ Granulosicoccus antarcticus (WP_088916663)
PdxJ Aureimonas sp. (WP_062011492)
PdxJ Aureimonas ureilytica (WP_019997951)
PdxJ Aureimonas frigidaquae (WP_062228816)
PdxJ Escherichia coli (AAA21845)
PdxJ Yersinia pestis (AAS62723)
PdxJ Pseudomonas putida (ADR61702)
0.5
B
PdxA Uncultured Desulfovibrio sp. (SCM70633)
PdxA Belnapia rosea (SDB68909)
PdxA Rhodoferax ferrireducens (WP_011465832)
PdxA Acidobacteriaceae bacterium (WP_020714026)
PdxA Acidiphilium spp. (WP_029311964)
PdxA Geminicoccus roseus (WP_027133679)
PdxA Acidocella sp. (WP_008495531)
PdxA Microvirga sp. (WP_036347039)
PdxA Burkholderia sp. (WP_013587875)
PdxA Burkholderia sp. (WP_018442029)
PdxA Herbaspirillum sp. (WP_007884029)
PdxA Paraburkholderia kururiensis (WP_081601894)
PdxA Burkholderia glumae (WP_015877567)
PdxA Paraburkholderia nodosa (WP_081771893)
PdxA Ralstonia solanacearum (CEJ17583)
PdxA Burkholderia sp. (WP_081922880)
PdxA Gammaproteobacteria bacterium (WP_035477203)
PdxA Pseudomonas asplenii (SDT30492)
PdxA Marinobacterium rhizophilum (WP_020680535)
PdxA Comamonas sp. (WP_021025226)
PdxA Burkholderia phymatum (pdb|4JQP)
PdxA Skermanella aerolata (WP_044425488)
PdxA Rhodospirillales bacterium (WP_088561829)
PdxA Novispirillum itersonii (WP_019644281)
PdxA Dolichoderus spp. symbiont JSC189
PdxA Dolichoderus spp. symbiont JSC188
PdxA Bartonella apis (WP_077990273)
PdxA Bacillus megaterium (KFN08701)
PdxA Nitratireductor basaltis (KFB10536)
PdxA Nitratireductor soli (WP_048647720)
PdxA Nitratireductor indicus (WP_009450244)
PdxA Nitratireductor aquibiodomus (WP_065818305)
PdxA Chelativorans sp. (WP_011581098)
PdxA Mesorhizobium sp. (WP_072603891)
PdxA Aminobacter sp. (WP_024848538)
PdxA Mesorhizobium sp. (WP_047143759)
PdxA Aliihoeflea sp. (WP_024586198)
PdxA Mesorhizobium alhagi (WP_085983927)
PdxA Aminobacter sp. (WP_055978698)
PdxA Pseudaminobacter salicylatoxidans (WP_019170540)
PdxA Mesorhizobium sp. (SFT74379)
PdxA Mesorhizobium spp. (WP_056109458)
PdxA Mesorhizobium spp. (WP_024926182)
PdxA Mesorhizobium sp. (WP_054310972)
PdxA Mesorhizobium plurifarium (CDX33853)
PdxA Aquamicrobium aerolatum (SFJ05471)
PdxA Mesorhizobium sp. (WP_027045366)
PdxA Mesorhizobium sp. (WP_023800700)
PdxA Mesorhizobium sp. (OJX83116)
PdxA Mesorhizobium sp. (WP_056102294)
PdxA Aquamicrobium defluvii (WP_035025963)
PdxA Pseudaminobacter manganicus (WP_080921871)
PdxA Mesorhizobium australicum (WP_085464882)
PdxA Rhizobiales bacterium (OJU05209)
PdxA Martelella sp. (AMM84706)
PdxA Martelella endophytica (WP_045682347)
PdxA Rhizobium sp. (WP_062375861)
PdxA Rhizobium sp. (WP_088682028)
PdxA Rhizobium tropici (WP_015339365)
PdxA Rhizobium sp. (OJU00716)
PdxA Phyllobacterium sp. (SDO97050)
PdxA Phyllobacterium sp. (SDP57273)
PdxA Phyllobacterium sp. (WP_008128512)
PdxA Paramesorhizobium deserti (WP_068881941)
PdxA Brucella suis (ABY37787)
PdxA Ochrobactrum intermedium (EEQ95352)
PdxA Ochrobactrum sp. (WP_078340732)
PdxA Rhizobiales bacterium (OJX99460)
PdxA Ochrobactrum pituitosum (WP_079211707)
PdxA Pseudochrobactrum sp. (WP_075654631)
PdxA Mesorhizobium sp. (WP_082005529)
PdxA Dolichoderus spp. symbiont JSC161
PdxA Dolichoderus spp. symbiont JSC085
PdxA Bartonella sp. (WP_078705171)
PdxA Bartonella clarridgeiae (WP_013544900)
PdxA Bartonella australis (WP_015397856)
PdxA Bartonella ancashensis (WP_071628371)
PdxA Bartonella bacilliformis (WP_042994835)
PdxA Bartonella queenslandensis (WP_039759891)
PdxA Bartonella rattaustraliani (WP_019223870)
PdxA Bartonella tamiae (WP_008039157)
PdxA Bartonella apis (WP_075870038)
PdxA Bartonella apis (AQT44587)
PdxA Escherichia coli (AAA24305)
PdxA Pseudomonas putida (ADR58134)
0.2
C
PdxK Ca. Tokpelaia hoelldoblerii (AQS40823)
PdxK Bartonella apis (WP_077973217)
PdxK Rhizobium sp. (WP_084365874)
PdxK Rhizobium gallicum (WP_039847051)
PdxK Rhizobium mesoamericanum (WP_007528649)
PdxK Rhizobium etli (WP_086082882)
PdxK Rhizobium spp. (WP_056324036)
PdxK Rhizobium arenae (WP_075291861)
PdxK Sinorhizobium fredii (WP_012709802)
PdxK Sinorhizobium spp. (WP_012067391)
PdxK Sinorhizobium saheli (WP_066876580)
PdxK Sinorhizobium sp. (WP_069060563)
PdxK Rhizobium sp. (WP_062577192)
PdxK Rhizobium sp. (WP_081952898)
PdxK Rhizobium sp. (WP_071007006)
PdxK Rhizobium marinum (WP_029617607)
PdxK Rhizobium undicola (WP_027488192)
PdxK Rhizobium sp. (WP_062557224)
PdxK Ochrobactrum pituitosum (WP_079212331)
PdxK Ochrobactrum cytisi (WP_071634389)
PdxK Phyllobacterium sp. (WP_091879563)
PdxK Phyllobacterium spp. (WP_027231058)
PdxK Phyllobacterium sp. (WP_008122066)
PdxK Mesorhizobium sp. (WP_047145587)
PdxK Mesorhizobium sp. (WP_095792194)
PdxK Mesorhizobium sp. (ODT08873)
PdxK Nitratireductor basaltis (WP_036485529)
PdxK Escherichia coli O104:H4 (EGT68447)
PdxK Pseudomonas fluorescens (AEV65834)
PdxK Klebsiella pneumoniae (CDO15728)
PdxK Salmonella enterica (ASF65309)
0.5

Table S1. Primers and probes used in this study.

Name	Sequence	Reference
FISH probes		
EUB338	5'- GCT GCC TCC CGT AGG AGT -3'	Łukasik *et al.* (2017)
EUB897	5′-TTT GAG TTT YAV YCT TGC G-3′	Łukasik *et al.* (2017)
Rhiz1244	5'- TCG CTG CCC ACT GTC ACC -3'	Thayanukul *et al*. (2010)
Primers for sequencing		
9Fa	5'- GAG TTT GAT CIT IGC TCA G -3'	Łukasik *et al* (2017); Russell *et al.* (2009)
1513R	5'- TAC IGI TAC CTT GTT ACG ACT T -3'	Russell *et al.* (2009)
789F	5'- TAG ATA CCC SSG TAG TCC -3'	Baker *et al*. (2003)
907R	5'- CCG TCA ATT CMT TTG AGT TT -3'	Russell *et al.* (2009)

Table S2. Length, codon adaptation index (CAI), GC3%, GC% and Blast hits (which BLAST, which e-value) for each contig in the assembled sequence data from the *Dolichoderus* spp. symbionts.

Dolichoderus spp. symbiont JSC085

Contig #	Length (bp)	Average CAI	Average GC3%	Average GC%	BlastN Hits					
					Accession number	Name	Identity %	Alignment length	E-value	Score
**1**	797173	0.224	0.335	0.434	CP017315	*Ca.* Tokpelaia hoelldobblerii	94.09	2655	0	2177
					FN645454.1	*Bartonella clarridgeiae* strain 73, complete genome	93.53	2629	0	2112
**2**	262201	0.228	0.346	0.441	CP017315	*Ca.* Tokpelaia hoelldobblerii	76.56	9523	0.0	2728
					CP003124.1	*Bartonella vinsonii* subsp. berkhoffii str. Winnie, complete genome	76.4	9528	0	2657
					BlastX Hit					
					Accession number	Name	Identity %	Alignment length	E-value	Score
**3**	19988	0.209	0.233	0.385	bhsal00710	Phage terminase, PBSX family large subunit [*Ca.* Tokpelaia hoelldobblerii]	59.36	438	0	1380
					WP_008041115.1	terminase [*Bartonella tamiae*]	63.87	429	0	1480

Dolichoderus spp. symbiont JSC161

Contig #	Length (bp)	Average CAI	Average GC3%	Average GC%	BlastN Hits					
					Accession number	Name	Identity %	Alignment length	E-value	Score
**1**	714118	0.201	0.248	0.385	CP017315	*Ca.* Tokpelaia hoelldobblerii	73.12	3043	0	555
					FN645459.1	*Bartonella rochalimae* ATCC BAA-1498 Contig5, whole genome shotgun sequence	78.96	4168	0	1510

**2**	122094	0.206	0.281	0.402	CP017315	*Ca.* Tokpelaia hoelldobblerii	93.23	2849	0.0	2259
					HG969192.1	*Bartonella tribocorum* main chromosome complete genome, strain BM1374166	92.04	2826	0	2136
**3**	102306	0.211	0.276	0.404	CP017315	*Ca.* Tokpelaia hoelldobblerii	80.11	181	2e-30	73
					FN645454.1	*Bartonella clarridgeiae* strain 73, complete genome	73.76	3361	0	662
**4**	20471	0.207	0.244	0.391	CP017315	*Ca.* Tokpelaia hoelldobblerii	84.45	328	3e-83	167
					FN645496.1	*Bartonella* sp. 1-1C contig11, whole genome shotgun sequence	74.44	1729	0	383
**6**	2169	No CDS			CP017315	*Ca.* Tokpelaia hoelldobblerii	94	1616	0.0	1318
					KP987881.1	Uncultured Rhizobiales bacterium 16S ribosomal RNA gene, complete sequence	95.57	1513	0	1309
					BlastX Hit					
					Accession number	Name	Identity %	Alignment length	E-value	Score
**5**	3056	0.156	0.280	0.362	bhsal06270	UTP-glucose-1-phosphate uridylyltransferase [*Ca.* Tokpelaia hoelldobblerii]	80.2	293	0	1259
					WP_051297171.1	hypothetical protein [Arsenophonus nasoniae]	66.67	174	3e-67	599

Dolichoderus spp. symbiont JSC188

Contig #	Length (bp)	Average CAI	Average GC3%	Average GC%	BlastN Hits					
					Accession number	Name	Identity %	Alignment length	E-value	Score
**1**	455897	0.224	0.303	0.413	CP017315	*Ca.* Tokpelaia hoelldobblerii	76.3	1810	0.0	514

					FN645454.1	*Bartonella clarridgeiae* strain 73, complete genome	76.37	2455	0	688
**2**	182740	0.212	0.298	0.405						
					CP017315	*Ca.* Tokpelaia hoelldobblerii	76.69	845	6e-128	249
					HG977196.1	*Bartonella schoenbuchensis* genomic scaffold, strain MVT06, scaffold 4	73.1	2695	0	494
**3**	108423	0.225	0.299	0.415						
					CP017315	*Ca.* Tokpelaia hoelldobblerii	95.6	91	1e-33	79
					FN645504.1	*Bartonella* sp. 1-1C contig19, whole genome shotgun sequence	77.62	1403	0	452
**4**	96938	0.227	0.298	0.407						
					CP017315	*Ca.* Tokpelaia hoelldobblerii	79.16	4247	0	1574
					HG977196.1	*Bartonella schoenbuchensis* genomic scaffold, strain MVT06, scaffold 4	79.75	4261	0	1639
**5**	89502	0.223	0.285	0.408						
					CP017315	*Ca.* Tokpelaia hoelldobblerii	89.3	187	3e-58	123
					FN645454.1	*Bartonella clarridgeiae* strain 73, complete genome	75.16	2858	0	688
**6**	84806	0.239	0.318	0.423						
					CP017315	*Ca.* Tokpelaia hoelldobblerii	83.46	387	1e-97	194
					BX897700.1	Bartonella quintana str. Toulouse, complete genome	76.54	5363	0	1533
**7**	83994	0.225	0.293	0.413						
					CP017315	*Ca.* Tokpelaia hoelldobblerii	96.47	85	4e-32	76
					FN645476.1	*Bartonella* sp. AR 15-3 contig9, whole genome shotgun sequence	75.7	1901	0	505
**8**	59636	0.227	0.283	0.412						
					CP017315	*Ca.* Tokpelaia hoelldobblerii	71.86	1148	3e-81	164
					HG969192.1	*Bartonella tribocorum* main chromosome complete genome, strain BM1374166	74.17	1440	1e-152	300
**9**	58802	0.219	0.285	0.401						

					CP017315	*Ca.* Tokpelaia hoelldobblerii	92.71	96	4e-31	74
					FN555106.1	*Bartonella birtlesii* genomic DNA locus for production of multiple variant pilus subunits	75.8	2603	0	685
**10**	52019	0.235	0.307	0.418						
					CP017315	*Ca.* Tokpelaia hoelldobblerii	93.71	3052	0.0	2457
					FN645466.1	*Bartonella rochalimae* ATCC BAA-1498 Contig12, whole genome shotgun sequence	90.39	3225	0	2261
**11**	20471	0.215	0.374	0.453						
					AJ422079.1	*Bartonella grahamii* cryptic plasmid pBRG1	94.74	38	4e-04	32
					BlastX Hits					
					Accession number	Name	Identity %	Alignment length	E-value	Score
**12**	5752	0.222	0.345	0.444						
					GAK34523.1	putative replication protein [alpha proteobacterium Q-1]	59.38	357	7e-134	1115

Dolichoderus spp. symbiont JSC189

Contig #	Length (bp)	Average CAI	Average GC3%	Average GC%	BlastN Hits					
					Accession number	Name	Identity %	Alignment length	E-value	Score
**1**	230662	0.237	0.357	0.435						
					CP017315	*Ca.* Tokpelaia hoelldobblerii	81.65	4295	0	1916
					CP014012.1	*Bartonella bacilliformis* strain ATCC:35685D-5, complete genome	80.38	4260	0	1718
**2**	177888	0.240	0.379	0.447						
					CP017315	*Ca.* Tokpelaia hoelldobblerii	79.63	1777	0	684
					FN645454.1	*Bartonella clarridgeiae* strain 73, complete genome	78.42	1997	0	689
**3**	129786	0.239	0.373	0.449						
					CP017315	*Ca.* Tokpelaia hoelldobblerii	95.56	2859	0	2474
					FN645454.1	*Bartonella clarridgeiae* strain 73, complete genome	93.22	2860	0	2268

**4**	126508	0.235	0.405	0.467						
					CP017315	*Ca.* Tokpelaia hoelldobblerii	77.72	1845	0	608
					CP000699.1	*Sphingomonas wittichii* RW1, complete genome	77.58	2346	0	752
**5**	88320	0.233	0.390	0.464						
					CP017315	*Ca.* Tokpelaia hoelldobblerii	77.69	2380	0	768
					CP003124.1	*Bartonella vinsonii* subsp. berkhoffii str. Winnie, complete genome	76.84	1170	0	351
**6**	76891	0.239	0.379	0.447						
					CP017315	*Ca.* Tokpelaia hoelldobblerii	78.08	2737	0	924
					HG969192.1	*Bartonella tribocorum* main chromosome complete genome, strain BM1374166	73.99	2684	0	571
**7**	71832	0.243	0.390	0.453						
					CP017315	*Ca.* Tokpelaia hoelldobblerii	76.64	993	6.00E-149	286
					CP009625.1	*Brucella abortus* 104M chromosome 1, complete sequence	77.13	1745	0	542
**8**	71235	0.231	0.364	0.444						
					CP017315	*Ca.* Tokpelaia hoelldobblerii	74.18	5984	0	1270
					CP003124.1	*Bartonella vinsonii* subsp. berkhoffii str. Winnie, complete genome	72.6	3197	0	524
**9**	65734	0.244	0.400	0.466						
					CP017315	*Ca.* Tokpelaia hoelldobblerii	77.92	1449	0	479
					LN998033.1	*Brucella* sp. F965 genome assembly BVF965, chromosome : 1	77.47	1429	0	453
**10**	62076	0.234	0.382	0.454						
					CP017315	*Ca.* Tokpelaia hoelldobblerii	74.59	2645	0	604
					CP008820.1	*Ochrobactrum anthropi* strain OAB chromosome 1, complete sequence	72.63	1509	5e-127	254
**11**	60991	0.238	0.375	0.447						
					CP017315	*Ca.* Tokpelaia hoelldobblerii	76.07	1755	0	482
					FN645454.1	*Bartonella clarridgeiae* strain 73, complete genome	74.36	2831	0	628

**12**	54894	0.232	0.367	0.439						
					CP017315	*Ca.* Tokpelaia hoelldobblerii	75.29	680	1e-84	170
					HG969191.1	*Bartonella henselae* complete genome, strain BM1374165	73.43	3455	0	663
**13**	47276	0.242	0.376	0.450						
					CP017315	*Ca.* Tokpelaia hoelldobblerii	76.8	2043	0	608
					KP987873.1	Uncultured Rhizobiales bacterium UvrB gene, complete cds	77.34	1889	0	596
**14**	44954	0.219	0.402	0.453						
					CP017315	*Ca.* Tokpelaia hoelldobblerii	76.96	1675	0	509
					CP000758.1	*Ochrobactrum anthropi* ATCC 49188 chromosome 1, complete sequence	74.27	1749	0	381
**15**	41744	0.236	0.368	0.440						
					CP017315	*Ca.* Tokpelaia hoelldobblerii	75.59	2061	0	530
					CP001562.1	*Bartonella grahamii* as4aup, complete genome	75.82	1588	0	419
**16**	25155	0.241	0.402	0.464						
					CP017315	*Ca.* Tokpelaia hoelldobblerii	96.34	82	6e-31	73
					CP016079.1	*Mesorhizobium loti* NZP2037, complete genome	73.58	1230	2e-123	247
**17**	18793	0.276	0.351	0.451						
					CP017315	*Ca.* Tokpelaia hoelldobblerii	80.3	5370	0	2161
					HG977196.1	*Bartonella schoenbuchensis* genomic scaffold, strain MVT06, scaffold 4	77.59	5373	0	1706
**18**	13869	0.233	0.468	0.492						
					CP016079.1	*Mesorhizobium loti* NZP2037, complete genome	75.18	1402	3e-178	345
**19**	13703	0.232	0.406	0.472						
					HG965802.1	*Bartonella henselae*, strain BM1374163 complete genome	75.8	566	1e-72	155
**20**	12852	0.241	0.418	0.450						
					CP010401.1	*Candidatus* Bartonella ancashi strain 20.00, complete genome	73.71	1381	2e-145	286
**22**	11255	0.212	0.381	0.438						
					CP017315	*Ca.* Tokpelaia hoelldobblerii	97.56	82	5e-33	76

					CP012156.1	Hyphomonadaceae bacterium UKL13-1, complete genome	97.47	79	4e-27	73
**23**	8858	0.212	0.397	0.451						
					CP017315	*Ca.* Tokpelaia hoelldobblerii	87.12	132	3e-35	80
					CP003123.1	*Bartonella australis* Aust/NH1, complete genome	86.43	140	4e-31	80
**24**	8460	0.267	0.400	0.467						
					FN645509.1	*Bartonella schoenbuchensis* R1 contig4, whole genome shotgun sequence	74.61	1154	4e-130	258
**25**	7613	0.204	0.365	0.452						
					EU795093.1	Uncultured bacterium ARCTIC09_G_06 genomic sequence	93.1	58	3e-12	46
**27**	6428	0.234	0.448	0.458						
					CP001562.1	*Bartonella grahamii* as4aup, complete genome	82.98	94	8e-12	45
**29**	4306	0.219	0.390	0.438						
					CP017315	*Ca.* Tokpelaia hoelldobblerii	76.13	331	3e-36	81
					CP003784.1	*Bartonella quintana* RM-11, complete genome	89.47	95	5e-22	63
BlastX Hits										
					Accession number	Name	Identity %	Alignment length	E-value	Score
**21**	11306	0.218	0.408	0.454						
					AQS41417.1	Conserved inner membrane protein [*Ca.* Tokpelaia hoelldobblerii]	47.37	76	9e-15	183
					GAK34523.1	putative replication protein [alpha proteobacterium Q-1]	66.67	348	1e-146	1222
**26**	7131	0.214	0.382	0.455						
					AQS41756.1	Phosphoenolpyruvate-protein phosphotransferase [*Ca.* Tokpelaia hoelldobblerii]	34.02	679	1e-83	752
					AGF75284.1	multiphosphoryl transfer protein FruB [*Bartonella vinsonii* subsp. berkhoffii str. Winnie]	58.12	671	0	1894
**28**	5901	0.222	0.385	0.470						
					AQS41872.1	Unique protein [*Ca.* Tokpelaia hoelldobblerii]	30.77	611	5e-55	530
					AEC18768.1	autotransporter [*Pusillimonas* sp. T7-7]	39.45	697	2e-104	979

**30**	4119	0.170	0.328	0.414	WP_053944002.1	repressor [*Candidatus* Bartonella ancashi]	52.17	138	4e-39	397
**31**	4107	0.193	0.400	0.462	WP_062943482.1	autotransporter outer membrane beta-barrel domain-containing protein [*Rhizobium leguminosarum*]	33.13	332	9e-30	351
**32**	3311	0.185	0.311	0.412	EJN15724.1	putative transcriptional regulator [*Bradyrhizobium* sp. YR681]	33.87	124	8e-13	196
**33**	2844	0.196	0.320	0.388	WP_036052200.1	transcriptional regulator [*Bradyrhizobium* sp. URHD0069]	31.63	98	9e-07	145
**34**	2656	0.224	0.351	0.424	YP_009221490.1	XRE family transcriptional regulator (endogenous virus) [*Sinorhizobium* phage phiLM21]	64.83	145	3e-57	526
**35**	2607	0.165	0.336	0.412	AQS42178.1	Unique protein 1 [*Ca.* Tokpelaia hoelldobblerii]	35.59	59	1e-04	89
					KPQ11776.1	hypothetical protein HLUCCO17_04685 [Rhizobiales bacterium HL-109]	38.54	96	2e-10	175
**36**	2178	0.250	0.389	0.452	AQS41754.1	N-acetylglucosamine-6-phosphate deacetylase [*Ca.* Tokpelaia hoelldobblerii]	70.75	359	0	1384
					EDT42052.1	N-acetylglucosamine-6-phosphate deacetylase [*Burkholderia ambifaria* MEX-5]	50.85	354	6e-113	922
**37**	1884	0.157	0.306	0.410	WP_054312108.1	transcriptional regulator [*Mesorhizobium* sp. 1M-11]	45.76	59	4e-06	136
**38**	981	0.234	0.388	0.476	WP_057850211.1	hypothetical protein, partial [*Bradyrhizobium valentinum*]	43.19	301	2e-50	483
**39**	974	0.246	0.423	0.394	No hit.					

Table S3. Genomes used in this study.

Species	Accession number
Bartonella species	
Bartonella australis strain NH1	NC_020300.1
Bartonella bacilliformis strain KC583	NC_008783.1
Bartonella birtlesii strain IBS325	NZ_CM001557.1
Bartonella clarridgeiae strain 73	NC_014932.1
Bartonella grahamii strain as4aup	
chromosome	NC_012846.1
plasmid pBGR3	NC_012847.1
Bartonella henselae strain Houston-1	NC_005956.1
Bartonella schoenbuchensis strain m07a	
chromosome scaffold 1	NZ_KB915627.1
scaffold 2	NZ_KB915628.1
scaffold 3	NZ_KB915629.1
plasmid pML	NZ_CM001845.1
plasmid pMS	NZ_CM001846.1
Bartonella tamiae strain Th239	
supercontig 1.1	NZ_JH725147.1
supercontig 1.2	NZ_JH725148.1
supercontig 1.3	NZ_JH725149.1
Bartonella tamiae strain Th307	
supercontig 1.1	NZ_JH725021.1
Bartonella tribocorum strain CIP105476	
chromosome	NC_010161.1
plasmid pBT	NC_010160.1

Bartonella vinsonii subsp. Berkhoffii strain Winnie	CP003124.1
Bartonella apis strain BBC0122	CP015625
Bartonella apis strain BBC0178	CP015820
Bartonella apis strain BBC0244	CP01515821
Tokpelaia species	
Ca. Tokpelaia holldobblerii	CP017315
Ca. Tokpelaia sp. strain JSC085	
Ca. Tokpelaia sp. strain JSC161	
Ca. Tokpelaia sp. strain JSC188	
Ca. Tokpelaia sp. strain JSC189	
Outgroups	
Agrobacterium tumefasciens strain C58	
chromosome I	NC_003062.2
chromosome II	NC_003063.2
plasmid pTi	NC_003065.3
plasmid pAt	NC_003064.2
Bradyrhizobium japonicum strain USDA110	
chromosome	NC_004463.1
Brucella melitensis strain 16M	
chromosome I	NC_003317.1
chromosome II	NC_003318.1
Mesorhizobium loti strain MAFF303099	
chromosome	NC_002678.2
plasmid pMLa	NC_002679.1
plasmid pMLb	NC_002682.1

Ochrobactrum anthropi strain ATCC49188	
chromosome 1	NC_009667.1
chromosome 2	NC_009668.1
plasmid pOANT01	NC_009669.1
plasmid pOANT02	NC_009670.1
plasmid pOANT03	NC_009671.1
plasmid pOANT04	NC_009672.1
Sinorhizobium meliloti strain 1021	
chromosome	NC_003047.1
plasmid pSymA	NC_003037.1
plasmid pSymB	NC_003078.1

Table S4. General genome data.

	Dolichoderus sp. symbiont JSC085	*Dolichoderus* sp. symbiont JSC161	*Dolichoderus* sp. symbiont JSC188	*Dolichoderus* sp. symbiont JSC189
Number of contigs	3	6	12	39
Maximal contig size (bp)	797173	714118	455897	230662
Contig N50	797173	714118	108423	76891
Total contig size (bp)	1079362	964214	1298980	1532052
Number of proteins	966	901	1073	1312
Coding %	85.1	91.1	78.6	74.4
Average gene length (bp)	951	975	952	869
RNA operons	1	1	1	1
tRNA	42	42	41	41
GC%	42.88	38.55	41.55	43.54
Completeness[1]	96.5%	98.3%	94.9%	92.0%

Table S5. BLASTx hits (e-value 10^{-10}) for intergenic regions.

***Dolichoderus* spp. symbiont JSC085**

Contig #	Position	Accession number	Best Blast Hit			
			Name	Identity %	E-value	Score
1	13351:13547	WP_068881977	DNA helicase RecG [Paramesorhizobium deserti]	70.59	2e-21	243
1	13833:14350	AQS41369	ATP-dependent DNA helicase RecG [Candidatus Tokpelaia hoelldoblerii]	61.21	2e-26	218
1	186110:186962	gbAIK44492	hypothetical protein DR92_1216 [Ochrobactrum anthropi]	65.93	9e-29	288
1	221709:221902	gbOJU83638	hypothetical protein BGO06_18820 [Shinella sp. 65-6]	66	7e-22	226
1	253464:254138	WP_018700794	hypothetical protein [Amorphus coralli]	36.84	2e-12	177
1	309767:310071	WP_078340837	di-trans,poly-cis-decaprenylcistransferase [Ochrobactrum sp. P6BS-III]	57.97	8e-17	205
1	350035:351262	WP_063951587	phosphoenolpyruvate carboxykinase (ATP) [Agrobacterium tumefaciens]	39.8	1e-24	289
1	354369:354927	CCG43388	conserved hypothetical protein [Phaeospirillum molischianum DSM 120]	69.35	1e-15	195
1	355387:360558	JAN94722	hypothetical protein, partial [Daphnia magna]	75.25	3e-71	638
1	378664:379103	WP_078339627	heat-inducible transcriptional repressor HrcA [Ochrobactrum sp. P6BS-III]	80.77	5e-29	213
1	509239:509581	WP_077969772	ABC transporter ATP-binding protein [Bartonella apis]	68	4e-14	193
1	542985:543421	WP_009504215	DNA polymerase I [Citreicella sp. 357]	35.83	2e-16	146

1	543650:544300	WP_008037833	DNA polymerase I [Bartonella tamiae]	43.43	5e-26	221
1	591154:591770	WP_019218786	ATPase [Bartonella florencae]	45.45	2e-14	117
1	651547:652274	SFN06071	glutamate/aspartate transport system ATP-binding protein [Variovorax sp. OV329]	85.1	2e-15	204
1	654994:656945	WP_083338225	phage portal protein [Ensifer sp. LCM 4579]	60.38	1e-16	144
1	736812:737156	CDX38255	protein chain initiation factor IF-3 [Mesorhizobium plurifarium]	70.97	3e-20	229
1	782144:782533	WP_013891272	tRNA (guanosine(46)-N7)-methyltransferase TrmB [Mesorhizobium opportunistum]	69.2	6e-26	271
1	802457:803391	CDX52279	hypothetical protein MPL1032_140306 [Mesorhizobium plurifarium]	63.64	1e-12	180
2	12373:12849	WP_011179679	glutathione S-transferase [Bartonella quintana]	63.46	2e-12	176
2	45878:46788	CDX11302	hypothetical protein MPLB_1090003 [Mesorhizobium sp. ORS3324]	88	2e-11	115

***Dolichoderus* spp. symbiont JSC161**

Contig #	Position	Accession number	Best Blast Hit			
			Name	Identity %	E-value	Score
1	82649:83030	CDX11302	hypothetical protein MPLB_1090003 [Mesorhizobium sp. ORS3324]	58.44	5e-16	204
1	205928:206410	CDX30154	hypothetical protein MPLDJ20_120761 [Mesorhizobium plurifarium]	52.7	1e-25	269

1	220597:222905	WP_075869694	protein ndvB [Bartonella apis]	42.95	1e-114	777
1	223308:224156	WP_041848944	protein ndvB [Bartonella bacilliformis]	38.6	1e-48	382
1	245466:245819	WP_075913738	hypothetical protein [Bartonella apis]	44.44	1e-12	171
1	280704:281116	WP_041848834	16S rRNA (uracil(1498)-N(3))-methyltransferase [Bartonella bacilliformis]	52.69	3e-23	253
1	399870:400110	WP_029030828	50S ribosomal protein L34 [Salinarimonas rosea]	84.09	6e-15	179
2	21608:714119	WP_026088061	23S rRNA methyltransferase [Bartonella rattaustraliani]	71.43	1e-22	241
2	22453:22940	EEW80204	translation initiation factor IF-3 [Brucella abortus NCTC 8038]	77.88	2e-47	417
2	106255:10443	WP_007873235	bifunctional uridylyltransferase/uridylyl-removing protein [Ochrobactrum sp. CDB2]	35.37	2e-41	211
2	118223:118614	SDP92290	hypothetical protein SAMN05443582_1226 [Phyllobacterium sp. OV277]	82	2e-20	223

Dolichoderus **spp. symbiont JSC188**

Contig #	Position	Accession number	Best Blast Hit			
			Name	Identity %	E-value	Score
1	69435:70750	WP_022710225	MULTISPECIES: L,D-transpeptidase [Pseudochrobactrum]	51.43	3e-23	269
1	163326:164254	WP_068881913	hypothetical protein [Paramesorhizobium deserti]	37.76	4e-13	154

1	219159:221258	CUX39133	hypothetical protein AGR6A_Cc60098 [Agrobacterium genomosp. 6 str. NCPPB 925]	69.23	8e-22	174
1	331704:332288	WP_083212959	exodeoxyribonuclease VII large subunit [Nitratireductor aquibiodomus]	60	1e-48	449
1	370256:370814	AQS41339	Hypothetical protein BHV28_06360 [Candidatus Tokpelaia hoelldoblerii]	51.22	2e-12	172
2	74149:76143	WP_077971287	ABC transporter substrate-binding protein [Bartonella apis]	40.98	5e-12	191
2	170611:171216	WP_024925246	MULTISPECIES: choline-sulfatase [Mesorhizobium]	89.91	2e-65	539
2	171877:173678	WP_035023086	choline-sulfatase [Aquamicrobium defluvii]	84.78	2e-44	227
3	102955:104152	WP_080824387	carbonate dehydratase [Agrobacterium genomosp. 6]	40.21	6e-16	157
4	94703:95140	SDP17579	Ppx/GppA phosphatase [Phyllobacterium sp. OV277]	56.06	7e-27	182
4	95925:96454	WP_056697818	MULTISPECIES: ABC transporter [Aureimonas]	87.8	3e-11	176
6	26317:27038	WP_022712468	DNA recombination protein RmuC [Pseudochrobactrum sp. AO18b]	60.53	1e-20	247
6	47349:48019	EEW80204	translation initiation factor IF-3 [Brucella abortus NCTC 8038]	77.78	2e-37	356
9	56597:57651	WP_008039041	porin [Bartonella tamiae]	50.63	7e-15	211
10	4899:10087	JAN94722	hypothetical protein, partial [Daphnia magna]	77.24	2e-58	378
10	10547:11038	SDP92290	hypothetical protein SAMN05443582_1226 [Phyllobacterium sp. OV277]	82	1e-19	221
11	9729:10517	WP_072572662	prevent-host-death protein [Granulibacter bethesdensis]	84	5e-35	333
11	17865:18985	WP_045727535	plasmid replication initiator-like protein [Xanthomonas sp. GPE 39]	62.3	4e-15	211

***Dolichoderus* spp. symbiont JSC189**

Contig #	Position	Accession number	Best blast Hit			
			Name	Identity %	E-value	score
1	23937:25437	WP_080918055	thioredoxin [Pseudaminobacter manganicus]	49.32	3e-12	191
1	48381:48840	YP_009322781	homing endonuclease [Salmonella phage IME207]	42.7	3e-14	166
1	50993:51342	WP_013350341	NrdH-redoxin [Glutamicibacter arilaitensis]	68.52	1e-19	216
1	51610:52249	WP_046118921	nuclease [Sinorhizobium sp. PC2]	58.33	5e-12	150
1	118896:119473	SDP86530	RNA-binding protein Hfq [Phyllobacterium sp. OV277]	52.5	2e-14	187
1	119792:120290	WP_011400171	precorrin-3B C(17)-methyltransferase [Hahella chejuensis]<>Precorrin-3B methylase [Hahella chejuensis KCTC 2396]	40	9e-19	225
1	153872:154919	WP_080917776	NADPH:quinone oxidoreductase [Pseudaminobacter manganicus]	44.76	7e-18	179
1	162296:164452	WP_010705163	RNA degradosome polyphosphate kinase [Bartonella vinsonii]	55.95	7e-44	215
1	180046:181119	AQT42922	thiosulfate/3-mercaptopyruvate sulfurtransferase [Bartonella apis]	32.89	3e-13	120
1	214008:215143	WP_077992363	MFS transporter [Bartonella apis]<>Cyanate permease [Bartonella apis]	41.44	1e-13	202
1	217026:218410	ODU09013	ATPase [Rubrivivax sp. SCN 71-131]	41.82	3e-21	269
1	219560:221447	WP_008040233	hypothetical protein [Bartonella tamiae]	42.62	6e-17	220
1	221880:222889	WP_077972458	hypothetical protein [Bartonella apis]<>Phage Tail Collar Domain [Bartonella apis]	38.36	6e-27	263

2	19514:20585	WP_070068491	xanthine permease XanP [Acinetobacter qingfengensis]	56.41	1e-23	209
2	20850:21506	WP_069266113	xanthine permease XanP [Paraburkholderia nodosa]	40.86	4e-28	169
2	96290:97769	WP_057143769	hypothetical protein [Mesorhizobium sp. Root695]	58.46	2e-13	201
2	100524:101725	AQS41099	ABC transporter ATP-binding domain [Candidatus Tokpelaia hoelldoblerii]	44.72	9e-17	221
2	117241:117840	WP_006146855	phosphopantothenoylcysteine decarboxylase [Brucella melitensis]	53.97	5e-15	174
2	130983:132560	CAK02254	phage-related protein [Bartonella tribocorum CIP 105476]	69.23	4e-49	296
2	142510:143769	WP_052026847	integrase [Rhodovulum sp. PH10]	48.98	4e-18	236
3	7454:8653	WP_075915328	hypothetical protein [Bartonella apis]	32.9	1e-18	235
3	9419:10056	EEW80204	translation initiation factor IF-3 [Brucella abortus NCTC 8038]	80.77	2e-40	374
3	23356:27159	BAV48086	Putative uncharacterized protein [Mesorhizobium loti]	88.8	1e-54	503
3	27553:28229	JAN94728	daphnid bacterial-ribosomal-RNA-like, possible HGT [Daphnia magna]	65.85	7e-57	495
3	28689:30252	SDP92290	hypothetical protein SAMN05443582_1226 [Phyllobacterium sp. OV277]	86	9e-19	229
3	30779:31476	WP_046794124	MULTISPECIES: 50S ribosomal protein L34 [Rhizobium/Agrobacterium group]	90.91	5e-15	190
3	39562:39818	AQS41816	Methionine synthase (B12-dependent) [Candidatus Tokpelaia hoelldoblerii]	68.8	4e-23	165
3	41556:41768	AQS41816	Methionine synthase (B12-dependent) [Candidatus Tokpelaia hoelldoblerii]	68.42	2e-23	169
3	46687:47320	SDO72320	ribosome-associated protein [Phyllobacterium sp. OV277]	68.49	8e-26	270
3	77491:79502	AQT47065	PAS/PAC sensor signal transduction histidine kinase [Bartonella apis]	31.89	7e-20	177

4	8750:9073	WP_008039870	cobalt-precorrin-5B (C(1))-methyltransferase [Bartonella tamiae]	75	5e-19	171
4	9503:10563	WP_021585993	MULTISPECIES: precorrin-4 C(11)-methyltransferase [Ochrobactrum]	61.1	2e-36	294
4	11076:11272	WP_014240683	cobyric acid synthase CobQ [Azospirillum brasilense]	55	2e-11	167
4	110906:13608	WP_063687242	serine hydroxymethyltransferase [Sphingomonas sp. NIC1]	62.26	1e-14	167
4	15531:17406	WP_016745822	MULTISPECIES: transposase [Sphingomonadaceae]	56.96	1e-12	187
4	18448:19225	AQT43698	Ser/Thr protein kinase RdoA involved in Cpx stress response, MazF antagonist [Bartonella apis]	49.32	3e-39	380
4	40814:41659	AQT46185	hypothetical protein BBC0122_000440 [Bartonella apis]	75.76	7e-12	125
4	51928:52839	WP_068880168	ribosomal subunit interface protein [Paramesorhizobium deserti]	62	6e-11	172
4	85179:85733	WP_006209750	hypothetical protein [Brucella abortus]	48.5	4e-45	200
4	124128:124318	CDH85457	hypothetical protein SMRU11_1257 [Sinorhizobium meliloti RU11/001]	54.24	3e-11	155
5	2214:3196	CUX10898	hypothetical protein AGR8A_Cc30297 [Agrobacterium fabrum str. J-07]	54.81	9e-27	294
5	51564:54905	AQS41724	Epoxyqueuosine reductase [Candidatus Tokpelaia hoelldoblerii]	83.52	1e-49	438
5	124011:124453	WP_068387497	cytosine methyltransferase [Ventosimonas gracilis]	53.95	5e-13	185
5	131675:133681	KYM05525	NADP-dependent oxidoreductase, partial [Klebsiella pneumoniae]	50.57	7e-17	223
6	75900:76710	WP_023828528	tail sheath protein [Mesorhizobium sp. L103C120A0]	75.36	1e-26	295
7	35321:36148	WP_076781514	hypothetical protein [Brucella sp. 09RB8471]	51.43	6e-13	179
7	36911:38036	CDP79846	hypothetical protein BN1046_00750 [Bartonella schoenbuchensis]	44.66	2e-20	240
8	68328:69145	ANN61566	integrase [Mesorhizobium loti NZP2037]	60.92	2e-20	247

10	16291:16643	SCB26419	cob(II)yrinic acid a,c-diamide reductase /5,6-dimethylbenzimidazole synthase [Rhizobium multihospitium]	56.86	6e-12	170
10	35919:36780	AQS40981	ABC transporter periplasmic component protein [Candidatus Tokpelaia hoelldoblerii]	44.23	2e-19	107
10	51214:51800	OJU36432	radical SAM protein [Rhizobiales bacterium 68-8]	49.37	4e-11	174
11	52382:52527	WP_014815148	cobalt transporter [Mycobacterium chubuense]	67.39	3e-11	157
15	1646:3217	WP_015398355	phage repressor protein [Bartonella australis]	41.05	2e-18	162
15	4311:5156	WP_085034028	hypothetical protein [Ensifer aridi]	50	5e-14	190
15	16973:17442	OJX99294	repressor LexA [Rhizobiales bacterium 63-22]	61.54	3e-13	136
15	21335:22206	WP_068391400	cytosine methyltransferase, partial [Ventosimonas gracilis]	52.83	4e-15	207
16	9416:10382	SDR35450	catalase [Pseudovibrio sp. Tun.PHSC04-5.I4]	43.33	1e-19	137
16	18702:21058	WP_085025582	terminase [Ensifer aridi]	43	2e-13	116

Table S7. 16S rRNA identities and the average amino acid identity (AAI) values for the *Dolichoderus* spp. symbionts and *Ca*. Tokpelaia hoelldobblerii.

	Dolichoderus sp. symbiont JSC085	*Dolichoderus* sp. symbiont JSC161	*Dolichoderus* sp. symbiont JSC188	*Dolichoderus* sp. symbiont JSC189	*Ca*. T. hoelldobblerii
Dolichoderus sp. symbiont JSC085		95.74/66.05	96.45/67.37	95.83/69.14	95.97/65.44
Dolichoderus sp. symbiont JSC161			95.54/64.67	95.33/65.63	95.61/62.5
Dolichoderus sp. symbiont JSC188				97.38/78.23	96.1/65.67
Dolichoderus sp. symbiont JSC189					96.64/62.38

Table S11. Lipopolysaccharide and peptidoglycan synthesis genes.

	Ca. T. hoelldobblerii	*Dolichoderus* spp. symbiont JSC085	*Dolichoderus* spp. symbiont JSC161	*Dolichoderus* spp. symbiont JSC188	*Dolichoderus* spp. symbiont JSC189
Bartonella lipopolysaccharide synthesis genes					
lpxA	bhsal07410	JSC085_contig_1_272	JSC161_contig_1_474	JSC188_contig_5_39	JSC189_contig_3_84
lpxB	bhsal07430	JSC085_contig_1_270	JSC161_contig_1_472	JSC188_contig_5_41	JSC189_contig_3_86
lpxC	bhsal12860	JSC085_contig_2_127	JSC161_contig_1_366	JSC188_contig_1_205	JSC189_contig_5_18
lpxD	bhsal07390	JSC085_contig_1_274	JSC161_contig_1_476	JSC188_contig_5_37	JSC189_contig_3_82
lpxK	bhsal13040	JSC085_contig_1_34	JSC161_contig_1_330	JSC188_contig_7_8	JSC189_contig_5_27
lpxL	bhsal09400	-	JSC161_contig_1_574	JSC188_contig_2_61	JSC189_contig_1_129
kdtA	bhsal13050	JSC085_contig_1_33	JSC161_contig_1_329	JSC188_contig_7_9	JSC189_contig_5_26
kdsA	bhsal06490	JSC085_contig_1_143	JSC161_contig_1_109	JSC188_contig_1_9	JSC189_contig_6_51
kdsB	bhsal02570	JSC085_contig_1_491	JSC161_contig_1_430	JSC188_contig_8_26	JSC189_contig_10_29
Bartonella peptidoglycan synthesis genes					
murA	bhsal14680	JSC085_contig_1_445	JSC161_contig_1_519	JSC188_contig_5_3	JSC189_contig_3_10
murB	bhsal12910	JSC085_contig_2_134	JSC161_contig_2_32	JSC188_contig_1_200	JSC189_contig_5_13
murC	bhsal12920	JSC085_contig_2_135	JSC161_contig_2_33	JSC188_contig_1_199	JSC189_contig_5_12
murD	bhsal12960	JSC085_contig_2_138	JSC161_contig_2_36	JSC188_contig_1_196	JSC189_contig_5_9
murE	bhsal12990	JSC085_contig_2_141	JSC161_contig_2_39	JSC188_contig_1_193	JSC189_contig_5_6

murF	bhsal12980	JSC085_contig_2_140	JSC161_contig_2_38	JSC188_contig_1_194	JSC189_contig_5_7
murG	bhsal12930	JSC085_contig_2_136	JSC161_contig_2_34	JSC188_contig_1_198	JSC189_contig_5_11
murJ	bhsal04520	JSC085_contig_1_163	JSC161_contig_2_108	JSC188_contig_1_367	JSC189_contig_2_61
mraY	bhsal12970	JSC085_contig_2_139	JSC161_contig_2_37	JSC188_contig_1_195	JSC189_contig_5_8
mrcA	bhsal01220<br>bhsal09190<br>bhsal13270	JSC085_contig_1_351<br>JSC085_contig_1_620	JSC161_contig_1_580	JSC188_contig_1_187	JSC189_contig_1_51
dacA	bhsal09030	-	-	-	-
ftsI	bhsal13000	JSC085_contig_2_142	JSC161_contig_2_40	JSC188_contig_1_192	JSC189_contig_5_5
bacA	bhsal10070	JSC085_contig_2_15	JSC161_contig_1_598	-	-
ddl	bhsal12900	JSC085_contig_2_133	JSC161_contig_1_370	JSC188_contig_1_203	JSC189_contig_5_14
mtgA	bhsal01060	-	-	-	-
